# Evolution and Spread of Y280-Lineage H9N2 Low Pathogenicity Avian Influenza Viruses in Korea, 2020–2023

**DOI:** 10.1155/tbed/8009335

**Published:** 2025-08-13

**Authors:** Andrew Y. Cho, Tae-Hyeon Kim, Heesu Lee, Sun-Hak Lee, Yeram Seo, Bitgoeul Kim, Chung-Young Lee, Sungsu Youk, Jung-Hoon Kwon, Chang-Seon Song, Dong-Hun Lee

**Affiliations:** ^1^Avian Disease Laboratory, College of Veterinary Medicine, Konkuk University, Seoul, Republic of Korea; ^2^Department of Microbiology, School of Medicine, Kyungpook National University, Daegu, Republic of Korea; ^3^Department of Microbiology, College of Medicine, Chungbuk National University, Cheongju, Republic of Korea; ^4^Laboratory of Veterinary Microbiology, College of Veterinary Medicine, Kyungpook National University, Daegu, Republic of Korea; ^5^Wildlife Health Laboratory, College of Veterinary Medicine, Konkuk University, Seoul, Republic of Korea

## Abstract

In 2020, Y280-lineage H9N2 low pathogenic avian influenza was detected in South Korea for the first time. Active surveillance from live bird markets (LBMs) and farms revealed the continuous circulation of Y280-lineage H9N2 viruses, replacing the previously indigenous Y439-lineage and becoming dominant in South Korea. Antigenic mapping showed a substantial antigenic difference between the Y280 and Y439-lineage viruses. Bayesian phylodynamic approach suggests the introduction of Y280-lineage viruses from China and revealed the frequent exchange of viruses between LBM and farms. The presence of mammalian adaptation markers and high binding affinity to human-type sialic acid receptor raise a concern on interspecies transmission of the viruses to mammals. These findings underscore the importance of continued genomic surveillance to monitor the evolution of H9N2 viruses and assessment of zoonotic potential.

## 1. Introduction

H9N2 subtype low pathogenicity avian influenza viruses (LPAIVs) have been circulating in poultry across Asia, the Middle East, and Africa resulting in great economic losses in the poultry industry [1, 2]. Furthermore, human cases of H9N2 LPAIV infection have been reported, raising public health concerns [3]. To date, H9N2 viruses have diverged into three major genetic lineages in poultry: G1, Y439, and Y280 (also classified as BJ94 and G9). The Y280-lineage has been found mostly in poultry in China and Southeast Asia including Vietnam, Cambodia, Myanmar, and Indonesia [1].

In 2020, Y280-lineage H9N2 LPAIVs were detected in live bird markets (LBMs) in South Korea for the first time [4, 5]. Thereafter, the Y280-lineage H9N2 LPAIVs have been reported across various sectors of the poultry industry in South Korea, including LBM, broilers, and layers, affecting considerable economic impact [6]. The emergence of the Y280-lineage H9N2 LPAIV in South Korea raised significant concerns regarding its impact on poultry industry and public health [4, 5]. In addition, since the LBMs have been identified as crucial epicenters for the evolution and recombination of H9N2 AIVs [2, 6–9], the infiltration of Y280-lineage H9N2 LPAIV into Korean LBMs has raised concerns about the potential emergence of novel reassortant strains or genotypes with increased pathogenicity and zoonotic potential [6, 9].

A vaccination policy has been implemented in the Korea poultry industry to control the previously circulating Y439-lineage H9N2 AIVs since 2007 [10], with a newly developed vaccine strain targeting the Y280-lineage H9N2 only recently introduced to the market in 2023. In this context, the genetic changes and distinction between the Y439-lineage and the Y280-lineage H9N2 AIVs warrant thorough investigation of their antigenicity. It is also imperative to elucidate the genomic differences between these lineages through comprehensive genetic sequencing and comparative analyses. Such insights will inform the development of tailored control measures and vaccination strategies to effectively control the spread of Y280-lineage H9N2 viruses.

## 2. Materials and Methods

### 2.1. Sample Collection and Virus Isolation

Since the detection of the newly introduced Y280-lineage H9N2 viruses, tissue sample were collected from both LBMs and poultry farms. Sample collection from LBMs were routinely done as active surveillance. The sample collection ranges from January 2019 to November 2023, resulting in a total duration of about 5 years. A total of 626 birds were purchased from six LBMs around South Korea. Sample collection from poultry farms were clinical sign-based, where farm owners suspecting the infection of avian influenza would bring in carcasses or sick birds to the avian disease laboratory of Konkuk University (*n* = 643). Trachea and cecal tonsil were chosen for virus isolation due to their routine use in AIV diagnostics and high viral load during infection. Birds were euthanized and tissue homogenates were prepared from pooled trachea and cecal tonsils collected from each bird by diluting the combined tissue homogenates 10% (w/v) with phosphate-buffered saline. The suspensions were centrifuged at 3000 rpm for 10 min, and the supernatants were filtered with 0.45 µm pore syringe filters. The filtrates were inoculated into 10-day-old specific-pathogen-free (SPF) embryonated chicken eggs (ECEs) [11]. Allantoic fluid was harvested from the inoculated SPF ECEs and total RNA was extracted using the RNeasy kit (QIAGEN, Hilden, Germany) according to the manufacturer's instructions. Hemagglutinin positive allantoic fluid samples were tested for influenza A virus by using qRT-PCR targeting the matrix gene [12].

### 2.2. Hemagglutination Inhibition (HI) Assay and Antigenic Cartography

Antigenic cartography has been suggested to be a credible predictor of vaccine efficacy [13, 14]. Previous studies have reported the correlation of genomic differences with antigenic difference [15–17]. A total of 20 H9N2 viruses were used to produce antiserum in SPF chickens (Table 1). Five birds per group were immunized with oil-emulsified whole virus-inactivated immunogen of each isolate twice in 6 weeks term. Antiserum of 20 antigens were collected from bleeding the immunized birds. All antisera were tested for HI assay with homologous antigens and subjected to cross HI assay with all the other heterologous antigens [18]. Out of the total 20 strains, 13 showed sufficient antibody level for further analysis with antigenic cartography. Cross HI assay results were then normalized and visualized as antigenic map with Racmacs program (https://acorg.github.io/Racmacs/) [13, 14, 19, 20]. All animal procedures conducted in this study were reviewed, approved, and supervised by the Institutional Animal Care and Use Committee (IACUC) of Konkuk University (KU21199). Antigenic sites based on the deduced amino acid sequences of Y280-lineage H9N2 viruses were examined to monitor the accumulation of amino acid mutations in the antigenic sites suggested previously [21].

### 2.3. Whole Genome Sequencing and Phylogenetic Analysis

For full-length genome sequencing, RNA extracted from allantoic fluids of all isolates confirmed as H9N2 subtype AIVs were amplified by one-step RT-PCR [22]. The RT-PCR amplicons (200 ng) of all eight gene segments was used to prepare the library for Illumina next-generation sequencing (NGS), according to the manufacturer's instructions. Illumina Miniseq (Illumina) was used to produce raw NGS data. De novo and directed assembly of genome sequences were carried out using the Geneious Prime software (http://www.geneious.com). We used A/H9 influenza virus clade classification tool (https://nmdc.cn/influvar/tools/H9aiv) developed by Fusaro et al. [23] to classify the viruses according to the newly proposed global classification and nomenclature, available on VarEPS-Influ (https://nmdc.cn/influvar/) [24]. Whole genome sequences were screened for novel mutations of mamalian marker using the FluSurver [25].

### 2.4. Bayesian Phylogenetic Analysis

The HA nucleotide sequences were used to infer the origin and the viral transmission of Y280 H9N2 viruses using Bayesian phylogenetic analysis using BEAST v1.10.4 [26–28]. Identical sequences (100% nucleotide sequence identity) were eliminated from the dataset. The HA sequences were assigned to three discrete traits according to the source of each virus: LBM, farm, and China. For an accurate reconstruction of the phylogeny, temporal signal of the HA sequences were examined using TempEst software with the maximum likelihood tree of the HA sequences constructed by RAxML v8 [27, 29]. The uncorrelated lognormal distribution relaxed clock method, the HKY nucleotide substitution model, and the GMRF Bayesian skyride coalescent prior were used for the flexible approach for inferring time-scaled phylogenetic analysis [30]. We reconstructed the ancestral state and estimated the asymmetric viral exchanges between the three different sources (LBM, *n* = 68; farm, *n* = 27; China, *n* = 8) using a nonreversible continuous-time Markov chain model. We applied a Bayesian stochastic search variable selection procedure to support the transition rate between host species statistically and to construct a Bayes factor (BF) test. Three independent Markov chains were executed to fulfill the criteria of an effective sampling size of > 200 as assessed by Tracer version 1.5 with a 10% burn-in (http://tree.bio.ed.ac.uk/software/tracer/). A maximum clade credibility (MCC) tree was generated using TreeAnnotator v1.10.4 (http://www.phylo.org/index.php/tools/treeannotator.html) and visualized with FigTree v1.4.2 (https://tree.bio.ed.ac.uk/). Viral transmission between discrete traits was visualized using SpreaD3 v0.9.7 software [31].

### 2.5. Molecular Markers of Y280-Lineage H9N2 Viruses

Whole genome sequences were used to deduce the amino acid sequences. Amino acid mutations associated with mammalian adaptation were screened using the FluSurver mutations app (http://flusurver.bii.a-star.edu.sg) and previous publications [32]. Amino acid mutations were investigated in comparison to those of the early Y280-lineage H9N2 viruses (A/Korean native chicken/South Korea/N20-84/2020, A/Korean native chicken/South Korea/N20-85/2020, A/Broiler/South Korea/N20-89/2020, A/Peckin duck/South Korea/N20-90/2020, A/Korean native chicken/South Korea/N20-98, and A/Korean native chicken/South Korea/N20-99/2020) reported in Korea to track any new addition to the previously reported mutations associated with mammalian adaptation [5].

### 2.6. Solid-Phase Sialic Acid Receptor Binding Assay

The receptor binding affinities of the Y280-like H9N2 virus were assessed using a solid-phase binding assay as previously described [33]. In brief, 96-well plates were coated with fetuin (Sigma-Aldrich, USA) and incubated with the A/chicken/Korea/SL20/2020(H9N2) (SL20) virus. After washing with PBST (PBS + 0.05% Tween 20) and blocking with PBS containing oseltamivir and desialylated BSA, biotin-labeled sialyglycopolymers (3′SLN-PAA-biot and 6′SLN-PAA-biot, glycotech, USA) were added to the wells and incubated for 1 h at 4°C. Following further washing, HRP-conjugated streptavidin was added and incubated. Finally, TMB substrate was used to develop the signal, the reaction was stopped with H_2_SO_4_, and absorbance at 450 nm was read.

## 3. Results

### 3.1. Surveillance and Whole Genome Sequencing

A total of 264 birds from LBMs and farms were tested in 2019, but no H9N2 LPAIV was detected in 2019. Y280-lineage H9N2 LPAIVs were consistently isolated every year after their first detection since 2020 (Table 2). A total of 159 H9N2 LPAIV isolates out of 626 sampled birds (25.4%) from LBM, and 29 H9N2 LPAIV isolates out of 643 samples (4.5%) from farm birds during 2020–2023 (Supporting Information 1).

For genome sequencing, the isolates were selected based on the collection date to reduce sampling bias and redundancy. A total of 57 and 22 whole genome sequences were acquired from the H9N2 virus isolates from LBMs and farms, respectively. All H9N2 isolates clustered with the Y280-lineage H9N2 viruses. No evidence of recombination was detected in any of the sequenced H9N2 virus genomes with other subtypes of AIV and among the Y280-lineage H9N2 (Figure S1). According to the new classification proposed by Fusaro et al. [23] the Y280-lineage H9N2 viruses in this study belonged to the clade B4.6.1.

### 3.2. Antigenic Cartography

**Figure 1:**
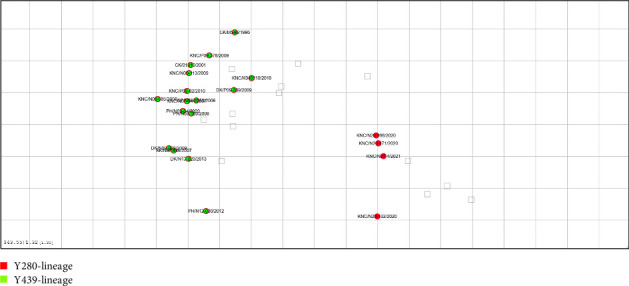
Antigenic cartography of H9N2 low pathogenic avian influenza viruses in Korea. Circles represent the position of the antigens (*n* = 20) in the map. Squares represent the position of the antiserums (*n* = 13) in the map. Y439-lineage H9N2 LPAIVs are colored in green. Y280-lineage H9N2 LPAIVs are colored in red. Each grid represents an antigenic unit (AU) which is twofold difference in HI titers between the strains.

Antigenic map of Y280 and Y439-lineage viruses each formed a cluster of each lineage (Figure 1). Y439-lineage showed more divergence among the viruses of their lineage (Table S3). No apparent trend between antigenic distance and time was observed within the Y439-lineage cluster (Figure 1). Each grid represents an antigenic unit (AU) which is twofold difference in HI titers between the strains. Within Y439-lineage cluster, the isolates exhibited antigenic differences up to 4 AU. Y280-lineage clustered more closely within their lineage. The seed strain of current inactivated Y280-lineage H9N2 vaccine in Korea had the highest nucleotide sequence identity (99.9%) to A/Korean native chicken/Korea/N20-132/2020 in this study. The difference between the Y439-lineage and Y280-lineage vaccine strain (A/chicken/Korea/01310/2001) was >6 AU. Currently circulating Y280-lineage viruses were observed to harbor low differences (within 2 AU) in antigenic sites from the early Y280-lineage viruses, with the greatest number of differences being limited to two mutations in the antigenic sites including the positions 189 and 190 which are located within the receptor binding site of HA protein (Table S2).

### 3.3. Phylodynamic Analysis of H9N2 Viruses

**Figure 2:**
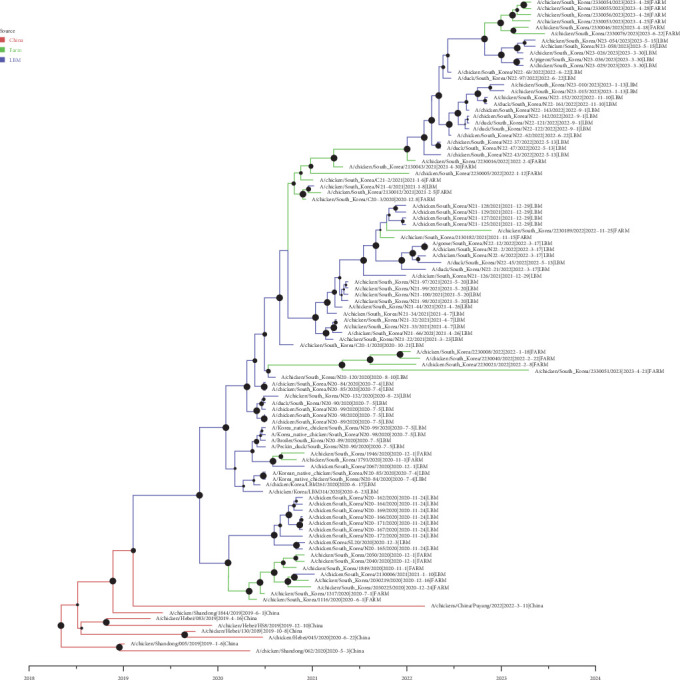
Maximum clade credibility tree constructed from Bayesian reconstruction of discrete trait analysis using the hemagglutinin genomes of Y280-lineage H9N2 low pathogenic avian influenza viruses, isolated from South Korea, 2020–2023. Branch colors represent each discrete trait assigned as shown upper left. Posterior probabilities are indicated by the size of black filled circles at each node. The horizontal axis represents each year as labeled.

Bayesian phylodynamic analysis with ancestral reconstruction of the Y280-lineage H9N2 revealed viral transmission from China to LBM in South Korea, subsequently disperse to poultry farm in South Korea (Figure 2). A single introduction of Y280-lineage H9N2 viruses fromChina to LBM in South Korea was detected with high statistical support ([BF] = 7.96, posterior probability [PP]: 0.87). The tMRCA of Korean isolates was August, 2019 (95% BCI: April 19, 2019 to November 27, 2019), indicating that Y280 virus was introduced into South Korea before August, 2019.

The frequent exchange of H9N2 viruses between poultry farms and LBMs in Korea was detected. At least six viral transmissions from LBMs to poultry farms were detected, followed by at least two instances of reintroduction from poultry farms back into the LBMs during August, 2020 (95% BCI: May, 2020 to October, 2020) and September, 2021 (95% BCI: June, 2021 to October, 2021). The exchange of viruses between LBM and farm sources was both highly statistically supported. The BF for the movement of Y280-lineage H9N2 virus from LBMs to farms (BF: 11,314, PP: 1.00) was higher than that of the from farms to LBMs (BF: 1413, PP: 1.00). Markov jump analysis revealed the frequency of viral movement of H9N2 from LBM to poultry farms (migration rate: 2.172, 95% BCI: 0.33 – 4.715) is almost as twice as likely than that of from poultry farms to LBM (migration rate: 1.218, 95% BCI: 0.066–2.882) (Table 3).

### 3.4. Mammalian Adaptation Molecular Markers

Amino acid sequences were deduced from the nucleotide sequences acquired from whole genome sequencing of Y280-lineage H9N2 LPAIVs. Mutations occurred throughout the circulation of Y280-lineage H9N2 LPAIVs in South Korea were examined (Table S1). All viruses had Q226L in HA which is known to play a critical role in increasing binding affinity to human-type sialic acid receptors. Transient and opportunistic mutations were found in fraction of isolates (Table 4), including A199T, K536R, A588V, and T598I in PB2, D622G in PB1, S37A, and K497R in PA, R167K in HA2, and N319S and S345G in NP which are associated with increased polymerase activity in mammalian cell lines or increased virulence in mice or ferrets ([7, 32, 35, 42, 47]. Mutations Q591K, E627K, and D701N in PB2 often associated with increased adaptation to mammals were not observed (Table 4). Mutations associated with drug resistance to neuraminidase inhibitors have been observed at I219V and S263R in NA [44, 45]. Mutations that affect the potential N-glycosylation site have been observed at S285N in HA and N66T and N83I in NA (Table 4).

**Figure 3:**
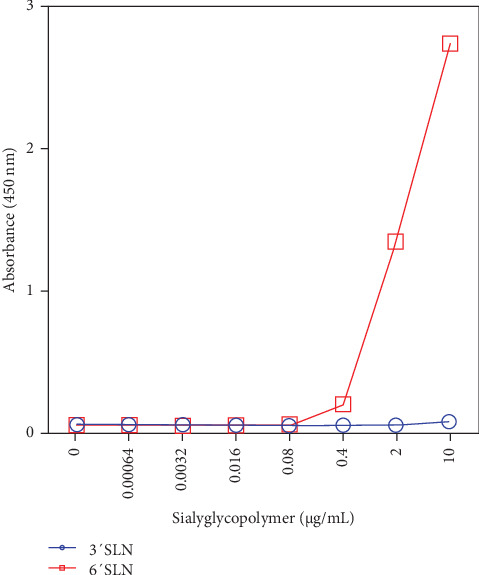
Receptor binding affinities to human- and avian-like receptors. Receptor binding avidities of H9N2 virus was measured using the solid-phase direct binding assay. Blue color indicates receptor binding avidities to avian-like receptor. Red color indicates receptor binding avidities to human-like receptor.

Avian influenza viruses generally show a stronger preference for α2,3-linked SA (sialic acid) over α2,6-linked SA, a characteristic considered a significant barrier to cross-species transmission into humans. The receptor binding specificity of a Y280-lineage H9N2 virus (SL20 strain) was measured using a solid-phase binding assay with biotinylated glycans containing either α2,3-linked SA or α2,6-linked SA. The Korean Y280-lineage H9N2 virus demonstrated a higher affinity for α2,6-linked SA compared to α2,3-linked SA (Figure 3). Consistent with previous studies in China, the Korean Y280-lineage H9N2 viruses have acquired a human-type receptor binding preference [48].

## 4. Discussion

Since its first report in 1996, Y439-lineage H9N2 LPAIVs had a significant impact to the poultry industry of South Korea. Extensive circulation of Y439-lineage H9N2 LPAIV had led to antigenic drift and generation of multiple reassortant H9N2 viruses [49]. It is noteworthy that no H9N2 LPAIV was isolated in 2019 in both LBM and farms in Korea, which highlights the disappearance of Y439-lineage H9N2 LPAIV in Korea [4, 8]. It seems that nationwide vaccination and surveillance effort have aided in the disappearance of Y439-lineage H9N2 LPAIV. However, the emergence and maintenance of Y280-lineage H9N2 LPAIVs in South Korea since 2020 pose significant risks to the poultry industry in South Korea and public health. Consistent with the findings of the previous study, the time of introduction from China to Korea was also estimated as early 2020.

Since the Y439-lineage H9N2 was first reported in 1996, at least five distinct genotypes have been detected up until 2016, possessing unique genome constellations resulting from reassortment with duck or wild bird LPAIVs. The first reassortant genotype K2 of Y439-lineage H9N2 LPAIV in South Korea was reported 7 years after the first report of Y439-lineage H9N2 in 1996 in South Korea [9]. The Y280-lineage H9N2 LPAIV introduced to South Korea in 2020 had identical genome constellation to the G57 genotype which have been circulating in Chinese poultry for an extended period, specifically belonging to the clade B4.6.1 [50]. As of the time this study was conducted, reassortment of Y280-lineage H9N2 LPAIV was not observed in the whole genome sequences analyzed in this study. The reassortment with the internal genes of Y280-lineage H9N2 LPAIV contributed to increase viral fitness in mammalian species, such as H7N9 viruses in China [50–52]. It is likely that selective pressures on the viral fitness of the Y280-lineage H9N2 LPAIV may be necessary to drive viral recombination, emphasizing the need for ongoing genomic surveillance to monitor its evolution.

Antigenic map revealed substantial distance between the Y280-lineage and Y439-lineage H9N2 viruses. As expected, the range of Y439-lineage cluster was larger than Y280-lineage as the Y439-lineage viruses had more time to evolve and diverge among the Korean poultry population, along with the vaccine pressure since 2007 [6]. A previous study reported an inverse correlation between protective efficacy and antigenic distance in H5N1 viruses, using sera from chickens and ferrets [53]. Also, in a H5N1 vaccine study, antigenic difference less than 4 AU was translated to a predictability of protection from vaccine [15, 17]. The antigenic difference of Y280-lineage from Y439-lineage H9N2 warrants the need to develop and implement vaccine with matching antigenicity [4, 5, 8, 54]. In this study, we confirmed that the antigenic difference between Y439 and Y280-lineage viruses exhibited more than 6 AU between the two vaccine seed strains. As of April 2023, a recombinant vaccine strain (rgHS314) using the glycoprotein of Y280-lineage H9N2 viruses and the backbone internal gene of A/Puerto Rico/8/34 (H1N1 and PR8) was developed and is on the market [55]. The implementation of the newly developed vaccine will help with the control of Y280-lineage H9N2 virus infection in poultry farms. Recent Y280-lineage viruses showed minimal antigenic differences based on the analysis of mutations in amino acid sequences of antigenic sites. Minimal mutations in the antigenic site may suggest minimal divergence in antigenicity [19]. Monitoring of antigenic escape variants against the vaccine will be paramount as they may arise and continue to circulate as shown before [49].

Bayesian phylogenetic analysis using the discrete trait analysis reconfirmed the introduction of the Y280-lineage H9N2 LPAIVs directly from China to LBM. This introduction was limited to a single incursion event, although the route of incursion still remains unclear [4, 9]. Thereafter, frequent exchange of the H9N2 viruses between LBM system and farms have been shown to expedite the evolution of the viruses [2, 6, 9, 52]. In this study, no reassortment events or novel mammalian markers have been identified since the initial analysis of the viruses reported by Youk et al.. Close monitoring of H9N2 viruses in both farms and LBM setting will be crucial to timely detect the emergence of novel viral strains with reassortment and mutations [5]. Source-sink dynamic analysis revealed a directional bias of virus transmission from LBM to farm, also supported by migration rate. High virus isolation rate from LBM samples may have reflected rampant and uncontrolled circulation of Y280-lineage H9N2 LPAIV [50, 56, 57]. This may be due to the continuous maintenance of viruses in the LBM setting, where a consistent supply of susceptible hosts is available. In contrast, farm poultries are raised in a rearing cycle and the viruses may not be maintained as well as it does in LBMs. However, we cannot rule out the possibility that the biased transmission resulted from sampling bias, as the number of LBM isolates was greater than that of poultry farm isolates.

Amino acid substitutions associated mammalian adaptation, new or removed potential N-glycosylation site, and resistance to neuraminidase inhibitors have been observed throughout the isolates sequenced in this study. However, key mutations frequently associated with mammalian adaptation, such as Q591K, E627K, and D701N in PB2, have not been observed. However, the Q226L mutation in the HA protein, known to play a crucial role in increasing binding affinity to human-type sialic acid receptors, has been maintained in Y280-lineage viruses, including our isolates, despite their long-term circulation in poultry species. This mutation and human-type receptor binding preference found in this study raise concerns on interspecies transmission of the viruses to mammals. Mutations associated with resistance to neuraminidase inhibitors could potentially have significant impact when combined with the propensity of Y280-lineage H9N2 LPAIVs to infect humans. Accumulation and combination of mutations could increase the ability of these viruses to infect humans, warranting the continued surveillance and tracking of the mutations through whole genome sequencing.

## 5. Conclusion

The findings from this study highlight the need to continue monitoring evolution of H9N2 viruses in both LBM and farms and performance of the current vaccine against the currently circulating H9N2 viruses. The introduction of the Y280-lineage H9N2 virus in South Korea highlights the complex interplay between viral evolution, host ecology, and public health. By examining the role of LBM and farms in viral transmission, investigating genetic and antigenic differences between viral lineages, and enhancing surveillance and control measures, we can mitigate the impact of H9N2 outbreaks and safeguard both poultry and human populations from the threat of avian influenza.

## Figures and Tables

**Table 1:** Viruses used to produce antigen and antiserum for cross-hemagglutination inhibition assay.

Strain name	Lineage	Year
A/chicken/Korea/MS96/1996	Y439	1996
A/chicken/Korea/01310/2001	Y439	2001
A/Guinea_fowl/Korea/N06-1259/2006	Y439	2006
A/Korean_native_chicken/Korea/N07-0341/2007	Y439	2007
A/Silkie_chicken/Korea/N07-0446/2007	Y439	2007
A/pheasant/Korea/N08-0180/2008	Y439	2008
A/Korean_native_chicken/Korea/N08-0585/2008	Y439	2008
A/white_peckin_duck/Korea/N09-002/2009	Y439	2009
A/pheasant/Korea/N09-064/2009	Y439	2009
A/Korean_native_chicken/Korea/N09-113/2009	Y439	2009
A/Korean_native_chicken/Korea/P09-378/2009	Y439	2009
A/white_peckin_duck/Korea/P09-389/2009	Y439	2009
A/Korean_native_chicken/Korea/K040110/2010	Y439	2010
A/Korean_native_chicken/Korea/P09-92/2010	Y439	2010
A/pheasant/Korea/N12-030/2012	Y439	2012
A/white_peckin_duck/Korea/N13-020/2013	Y439	2013
A/Korean_native_chicken/Korea/N20-132/2020	Y280	2020
A/Korean_native_chicken/Korea/N20-171/2020	Y280	2020
A/Korean_native_Chicken/Korea/N20-166/2020	Y280	2020
A/Korean_native_chicken/Korea/N21-4/2021	Y280	2021

**Table 2:** Surveillance of Y280-lineage H9N2 viruses from live bird markets and farms of South Korea.

Year	LBM sample collection	AIV positive from LBMs (isolation rate [%])	Farm sample collection	AIV positive from farms (isolation rate [%])
2019	74	0 (0)	190	0 (0)
2020	178	19 (10.6)	104	1 (0.96)
2021	130	16 (12.3)	134	6 (4.4)
2022	167	98 (58.7)	123	13 (10.6)
2023	77	26 (33.8)	92	9 (9.8)
Total	626	159 (25.4)	643	29 (4.5)

Abbreviation: LBMs, live bird markets.

**Table 3:** Bayes factor and statistical support value of discrete trait phylodynamic analysis of H9N2 viruses in South Korea.

Migration between discrete traits^a^		Actual migration rate	Bayes factor	Posterior probability
From	To			
LBM	Farm	2.172	11,314.88	1.00
Farm	LBM	1.218	1413.29	1.00
China	LBM	0.430	7.96	0.87

^a^Discrete trait “China,” “LBM,” and “farm” each represents source and sink of the viruses (“China”: viruses from China, “LBM”: viruses from live bird markets in South Korea, and “farm”: viruses from domestic chicken farms in South Korea).

**Table 4:** Frequency of molecular markers of Y280-lineage H9N2 LPAIV in South Korea associated with mammalian adaptation.

Protein	Mutation	Frequency	Effect	Reference
PB2	I63V	(3/55)	Decreased pathogenicity in mice	[34]
PB2	A199T	(1/55)	Increased virulence in mice	[35]
PB2	K526R	(5/55)	Determinants of pathogenicity of duck H5N1 viruses	[36]
PB2	A588V	(55/55)	Increased polymerase activity and virulence in mice	[32]
PB2	Q591K	(0/55)	Increased polymerase activity and virulence in mice	[32]
PB2	T598I	(9/55)	Increased polymerase activity and virulence in mice	[37]
PB2	E627K	(0/55)	Increased polymerase activity and virulence in mice	[32]
PB2	D701N	(0/55)	Increased polymerase activity and virulence in mice	[32]
PB1	D622G	(1/55)	Increased polymerase activity and virulence in mice	[38]
PA	S37A	(1/55)	Increased polymerase activity in mammalian cell line	[39]
PA	K497R	(1/55)	Increased polymerase activity in mammalian cell line	[40]
HA^b^	Q226L	(55/55)	Increased virus binding to α2–6	[32]
HA	S276N	(3/55)	Create new potential N-glycosylation site	FluSurver^a^
HA2	R167K	(8/55)	Increased virus binding to α2–6	[41]
NP	N319S	(11/55)	Increased polymerase activity and replication in mammalian cell line	[42]
NP	S345G	(6/55)	Associated with increased replication in ferret	[43]
NA	N66T	(8/55)	Remove potential N-glycosylation site	FluSurver
NA	N83I	(1/55)	Remove potential N-glycosylation site	FluSurver
NA	I219V	(1/55)	Strong drug resistance to oseltamivir and peramivir	[44]
NA	S263R	(3/55)	Strong drug resistance to oseltamivir and zanamivir	[45]
NS1	P87S	(1/55)	Host-specificity marker by statistics P:avian S:human	[46]

^a^N-glycosylation site prediction by FluSurver.

^b^HA is in H3 numbering.

## Data Availability

The data that support the findings of this study are openly available in NCBI GenBank at https://www.ncbi.nlm.nih.gov/genbank/, Reference Number PQ780784-PQ781215.

## Supporting Information

Supporting Information 1 Material 1. Complete sample collection record used in this study.

Supporting Information 2 Figure S1. Maximum-likelihood tree constructed using the (A) PB2, (B) PB1, (C) PA, (D) HA, (E) NP, (F) NA, (G) MP, and (H) NS gene of H9N2 isolated in this study. Bootstrap support of 70% or higher were considered well-supported and showed. Scale bar indicates nucleotide substitutions per site.

Supporting Information 3 Table S3. Cross-hemagglutination inhibition assay of H9N2 AIVs from South Korea.

Supporting Information 4 Table S2. Amino acid substitutions in antigenic sites of hemagglutinin protein sequences of each Y280-lineage H9N2 LPAIV isolate in order of time of isolation.

Supporting Information 5 Table S1. Amino acid substitutions associated with mammalian adaptation of each Y280-lineage H9N2 LPAIV isolates in order of time of isolation. Interpunct indicates that the deduced amino acid at the position possesses the same amino acid as that of the viruses reported previously by our group.
