# Supplementary material for: Evolution and Spread of Y280-Lineage H9N2 Low Pathogenicity Avian Influenza Viruses in Korea, 2020–2023
Source: Transbound Emerg Dis. 2025 Aug 13;2025:8009335. doi: 10.1155/tbed/8009335 (PMC12367370; doi:10.1155/tbed/8009335)
Supplement: Supporting Information 2 — Figure S1. Maximum-likelihood tree constructed using the (A) PB2, (B) PB1, (C) PA, (D) HA, (E) NP, (F) NA, (G) MP, and (H) NS gene of H9N2 isolated in this study. Bootstrap support of 70% or higher were considered well-supported and showed. Scale bar indicates nucleotide substitutions per site. [file 8009335.f2.pdf]

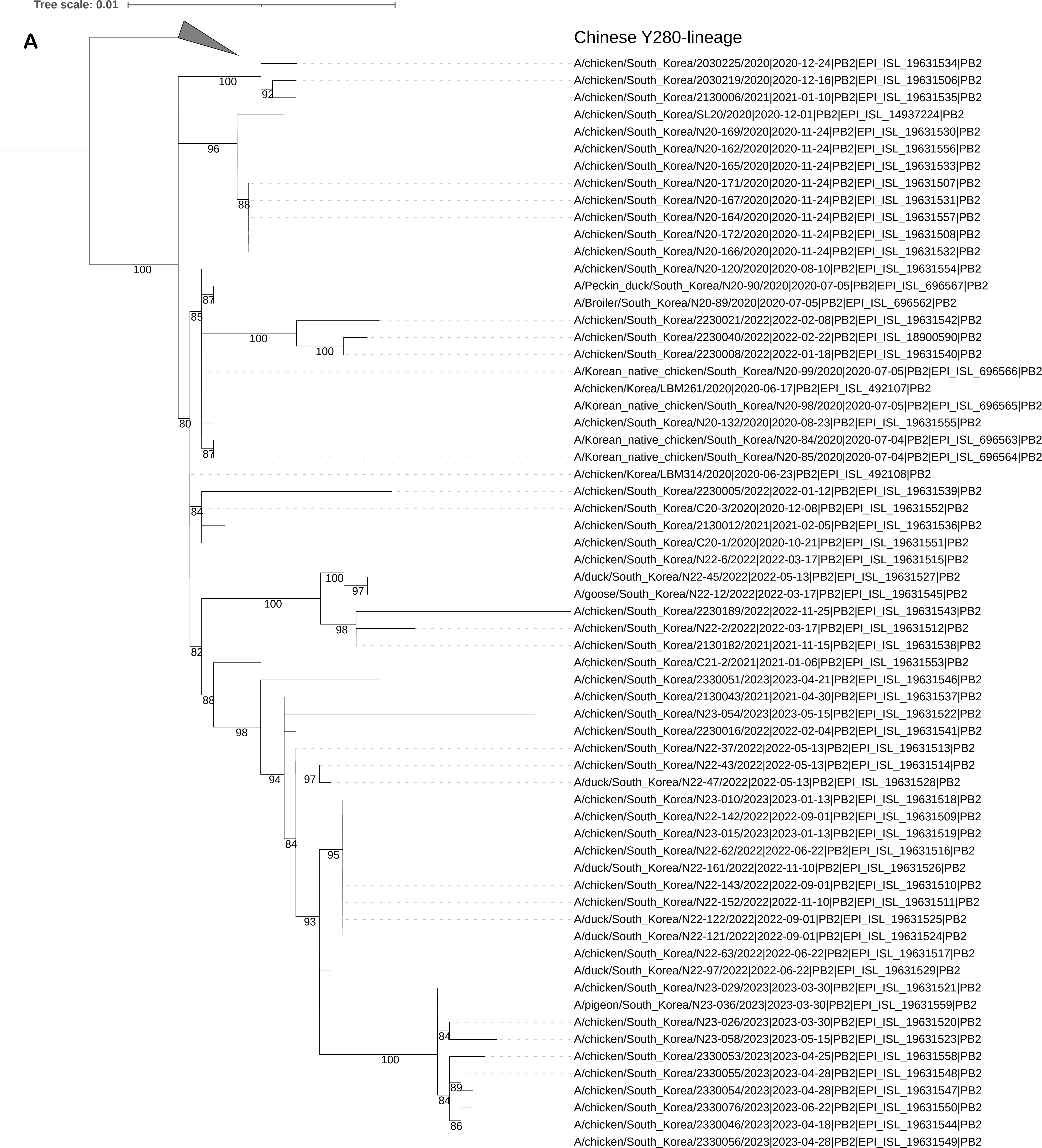
Tree scale: 0.01
A
Chinese Y280-lineage
A/chicken/South_Korea/2030225/2020|2020-12-24|PB2|EPI_ISL_19631534|PB2
A/chicken/South_Korea/2030219/2020|2020-12-16|PB2|EPI_ISL_19631506|PB2
A/chicken/South_Korea/2130006/2021|2021-01-10|PB2|EPI_ISL_19631535|PB2
A/chicken/South_Korea/SL20/2020|2020-12-01|PB2|EPI_ISL_14937224|PB2
A/chicken/South_Korea/N20-169/2020|2020-11-24|PB2|EPI_ISL_19631530|PB2
A/chicken/South_Korea/N20-162/2020|2020-11-24|PB2|EPI_ISL_19631556|PB2
A/chicken/South_Korea/N20-165/2020|2020-11-24|PB2|EPI_ISL_19631533|PB2
A/chicken/South_Korea/N20-171/2020|2020-11-24|PB2|EPI_ISL_19631507|PB2
A/chicken/South_Korea/N20-167/2020|2020-11-24|PB2|EPI_ISL_19631531|PB2
A/chicken/South_Korea/N20-164/2020|2020-11-24|PB2|EPI_ISL_19631557|PB2
A/chicken/South_Korea/N20-172/2020|2020-11-24|PB2|EPI_ISL_19631508|PB2
A/chicken/South_Korea/N20-166/2020|2020-11-24|PB2|EPI_ISL_19631532|PB2
A/chicken/South_Korea/N20-120/2020|2020-08-10|PB2|EPI_ISL_19631554|PB2
A/Peckin_duck/South_Korea/N20-90/2020|2020-07-05|PB2|EPI_ISL_696567|PB2
A/Broiler/South_Korea/N20-89/2020|2020-07-05|PB2|EPI_ISL_696562|PB2
A/chicken/South_Korea/2230021/2022|2022-02-08|PB2|EPI_ISL_19631542|PB2
A/chicken/South_Korea/2230040/2022|2022-02-22|PB2|EPI_ISL_18900590|PB2
A/chicken/South_Korea/2230008/2022|2022-01-18|PB2|EPI_ISL_19631540|PB2
A/Korean_native_chicken/South_Korea/N20-99/2020|2020-07-05|PB2|EPI_ISL_696566|PB2
A/chicken/Korea/LBM261/2020|2020-06-17|PB2|EPI_ISL_492107|PB2
A/Korean_native_chicken/South_Korea/N20-98/2020|2020-07-05|PB2|EPI_ISL_696565|PB2
A/chicken/South_Korea/N20-132/2020|2020-08-23|PB2|EPI_ISL_19631555|PB2
A/Korean_native_chicken/South_Korea/N20-84/2020|2020-07-04|PB2|EPI_ISL_696563|PB2
A/Korean_native_chicken/South_Korea/N20-85/2020|2020-07-04|PB2|EPI_ISL_696564|PB2
A/chicken/Korea/LBM314/2020|2020-06-23|PB2|EPI_ISL_492108|PB2
A/chicken/South_Korea/2230005/2022|2022-01-12|PB2|EPI_ISL_19631539|PB2
A/chicken/South_Korea/C20-3/2020|2020-12-08|PB2|EPI_ISL_19631552|PB2
A/chicken/South_Korea/2130012/2021|2021-02-05|PB2|EPI_ISL_19631536|PB2
A/chicken/South_Korea/C20-1/2020|2020-10-21|PB2|EPI_ISL_19631551|PB2
A/chicken/South_Korea/N22-6/2022|2022-03-17|PB2|EPI_ISL_19631515|PB2
A/duck/South_Korea/N22-45/2022|2022-05-13|PB2|EPI_ISL_19631527|PB2
A/goose/South_Korea/N22-12/2022|2022-03-17|PB2|EPI_ISL_19631545|PB2
A/chicken/South_Korea/2230189/2022|2022-11-25|PB2|EPI_ISL_19631543|PB2
A/chicken/South_Korea/N22-2/2022|2022-03-17|PB2|EPI_ISL_19631512|PB2
A/chicken/South_Korea/2130182/2021|2021-11-15|PB2|EPI_ISL_19631538|PB2
A/chicken/South_Korea/C21-2/2021|2021-01-06|PB2|EPI_ISL_19631553|PB2
A/chicken/South_Korea/2330051/2023|2023-04-21|PB2|EPI_ISL_19631546|PB2
A/chicken/South_Korea/2130043/2021|2021-04-30|PB2|EPI_ISL_19631537|PB2
A/chicken/South_Korea/N23-054/2023|2023-05-15|PB2|EPI_ISL_19631522|PB2
A/chicken/South_Korea/2230016/2022|2022-02-04|PB2|EPI_ISL_19631541|PB2
A/chicken/South_Korea/N22-37/2022|2022-05-13|PB2|EPI_ISL_19631513|PB2
A/chicken/South_Korea/N22-43/2022|2022-05-13|PB2|EPI_ISL_19631514|PB2
A/duck/South_Korea/N22-47/2022|2022-05-13|PB2|EPI_ISL_19631528|PB2
A/chicken/South_Korea/N23-010/2023|2023-01-13|PB2|EPI_ISL_19631518|PB2
A/chicken/South_Korea/N22-142/2022|2022-09-01|PB2|EPI_ISL_19631509|PB2
A/chicken/South_Korea/N23-015/2023|2023-01-13|PB2|EPI_ISL_19631519|PB2
A/chicken/South_Korea/N22-62/2022|2022-06-22|PB2|EPI_ISL_19631516|PB2
A/duck/South_Korea/N22-161/2022|2022-11-10|PB2|EPI_ISL_19631526|PB2
A/chicken/South_Korea/N22-143/2022|2022-09-01|PB2|EPI_ISL_19631510|PB2
A/chicken/South_Korea/N22-152/2022|2022-11-10|PB2|EPI_ISL_19631511|PB2
A/duck/South_Korea/N22-122/2022|2022-09-01|PB2|EPI_ISL_19631525|PB2
A/duck/South_Korea/N22-121/2022|2022-09-01|PB2|EPI_ISL_19631524|PB2
A/chicken/South_Korea/N22-63/2022|2022-06-22|PB2|EPI_ISL_19631517|PB2
A/duck/South_Korea/N22-97/2022|2022-06-22|PB2|EPI_ISL_19631529|PB2
A/chicken/South_Korea/N23-029/2023|2023-03-30|PB2|EPI_ISL_19631521|PB2
A/pigeon/South_Korea/N23-036/2023|2023-03-30|PB2|EPI_ISL_19631559|PB2
A/chicken/South_Korea/N23-026/2023|2023-03-30|PB2|EPI_ISL_19631520|PB2
A/chicken/South_Korea/N23-058/2023|2023-05-15|PB2|EPI_ISL_19631523|PB2
A/chicken/South_Korea/2330053/2023|2023-04-25|PB2|EPI_ISL_19631558|PB2
A/chicken/South_Korea/2330055/2023|2023-04-28|PB2|EPI_ISL_19631548|PB2
A/chicken/South_Korea/2330054/2023|2023-04-28|PB2|EPI_ISL_19631547|PB2
A/chicken/South_Korea/2330076/2023|2023-06-22|PB2|EPI_ISL_19631550|PB2
A/chicken/South_Korea/2330046/2023|2023-04-18|PB2|EPI_ISL_19631544|PB2
A/chicken/South_Korea/2330056/2023|2023-04-28|PB2|EPI_ISL_19631549|PB2

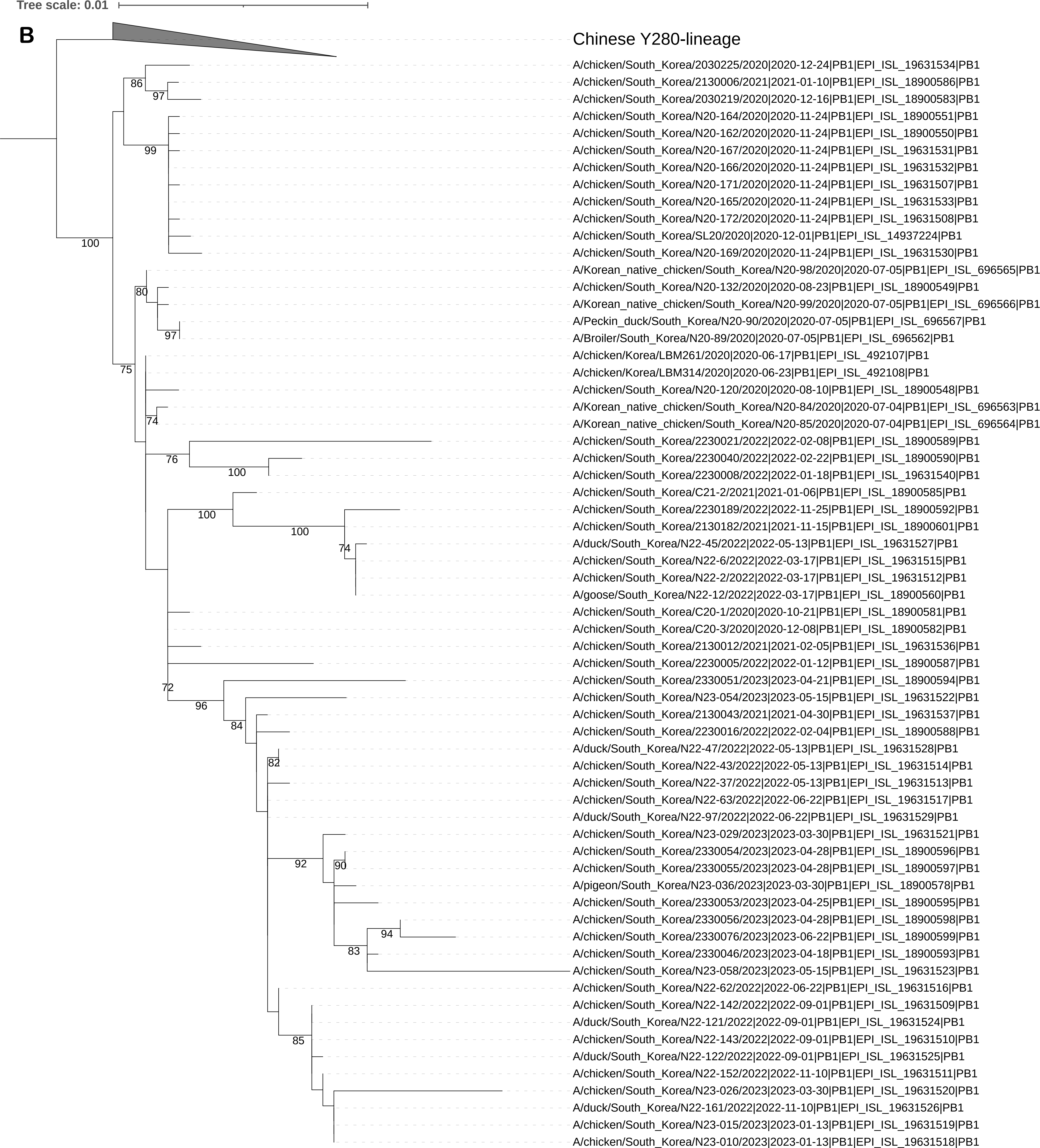
Tree scale: 0.01
B
Chinese Y280-lineage
A/chicken/South_Korea/2030225/2020|2020-12-24|PB1|EPI_ISL_19631534|PB1
A/chicken/South_Korea/2130006/2021|2021-01-10|PB1|EPI_ISL_18900586|PB1
A/chicken/South_Korea/2030219/2020|2020-12-16|PB1|EPI_ISL_18900583|PB1
A/chicken/South_Korea/N20-164/2020|2020-11-24|PB1|EPI_ISL_18900551|PB1
A/chicken/South_Korea/N20-162/2020|2020-11-24|PB1|EPI_ISL_18900550|PB1
A/chicken/South_Korea/N20-167/2020|2020-11-24|PB1|EPI_ISL_19631531|PB1
A/chicken/South_Korea/N20-166/2020|2020-11-24|PB1|EPI_ISL_19631532|PB1
A/chicken/South_Korea/N20-171/2020|2020-11-24|PB1|EPI_ISL_19631507|PB1
A/chicken/South_Korea/N20-165/2020|2020-11-24|PB1|EPI_ISL_19631533|PB1
A/chicken/South_Korea/N20-172/2020|2020-11-24|PB1|EPI_ISL_19631508|PB1
A/chicken/South_Korea/SL20/2020|2020-12-01|PB1|EPI_ISL_14937224|PB1
A/chicken/South_Korea/N20-169/2020|2020-11-24|PB1|EPI_ISL_19631530|PB1
A/Korean_native_chicken/South_Korea/N20-98/2020|2020-07-05|PB1|EPI_ISL_696565|PB1
A/chicken/South_Korea/N20-132/2020|2020-08-23|PB1|EPI_ISL_18900549|PB1
A/Korean_native_chicken/South_Korea/N20-99/2020|2020-07-05|PB1|EPI_ISL_696566|PB1
A/Peckin_duck/South_Korea/N20-90/2020|2020-07-05|PB1|EPI_ISL_696567|PB1
A/Broiler/South_Korea/N20-89/2020|2020-07-05|PB1|EPI_ISL_696562|PB1
A/chicken/Korea/LBM261/2020|2020-06-17|PB1|EPI_ISL_492107|PB1
A/chicken/Korea/LBM314/2020|2020-06-23|PB1|EPI_ISL_492108|PB1
A/chicken/South_Korea/N20-120/2020|2020-08-10|PB1|EPI_ISL_18900548|PB1
A/Korean_native_chicken/South_Korea/N20-84/2020|2020-07-04|PB1|EPI_ISL_696563|PB1
A/Korean_native_chicken/South_Korea/N20-85/2020|2020-07-04|PB1|EPI_ISL_696564|PB1
A/chicken/South_Korea/2230021/2022|2022-02-08|PB1|EPI_ISL_18900589|PB1
A/chicken/South_Korea/2230040/2022|2022-02-22|PB1|EPI_ISL_18900590|PB1
A/chicken/South_Korea/2230008/2022|2022-01-18|PB1|EPI_ISL_19631540|PB1
A/chicken/South_Korea/C21-2/2021|2021-01-06|PB1|EPI_ISL_18900585|PB1
A/chicken/South_Korea/2230189/2022|2022-11-25|PB1|EPI_ISL_18900592|PB1
A/chicken/South_Korea/2130182/2021|2021-11-15|PB1|EPI_ISL_18900601|PB1
A/duck/South_Korea/N22-45/2022|2022-05-13|PB1|EPI_ISL_19631527|PB1
A/chicken/South_Korea/N22-6/2022|2022-03-17|PB1|EPI_ISL_19631515|PB1
A/chicken/South_Korea/N22-2/2022|2022-03-17|PB1|EPI_ISL_19631512|PB1
A/goose/South_Korea/N22-12/2022|2022-03-17|PB1|EPI_ISL_18900560|PB1
A/chicken/South_Korea/C20-1/2020|2020-10-21|PB1|EPI_ISL_18900581|PB1
A/chicken/South_Korea/C20-3/2020|2020-12-08|PB1|EPI_ISL_18900582|PB1
A/chicken/South_Korea/2130012/2021|2021-02-05|PB1|EPI_ISL_19631536|PB1
A/chicken/South_Korea/2230005/2022|2022-01-12|PB1|EPI_ISL_18900587|PB1
A/chicken/South_Korea/2330051/2023|2023-04-21|PB1|EPI_ISL_18900594|PB1
A/chicken/South_Korea/N23-054/2023|2023-05-15|PB1|EPI_ISL_19631522|PB1
A/chicken/South_Korea/2130043/2021|2021-04-30|PB1|EPI_ISL_19631537|PB1
A/chicken/South_Korea/2230016/2022|2022-02-04|PB1|EPI_ISL_18900588|PB1
A/duck/South_Korea/N22-47/2022|2022-05-13|PB1|EPI_ISL_19631528|PB1
A/chicken/South_Korea/N22-43/2022|2022-05-13|PB1|EPI_ISL_19631514|PB1
A/chicken/South_Korea/N22-37/2022|2022-05-13|PB1|EPI_ISL_19631513|PB1
A/chicken/South_Korea/N22-63/2022|2022-06-22|PB1|EPI_ISL_19631517|PB1
A/duck/South_Korea/N22-97/2022|2022-06-22|PB1|EPI_ISL_19631529|PB1
A/chicken/South_Korea/N23-029/2023|2023-03-30|PB1|EPI_ISL_19631521|PB1
A/chicken/South_Korea/2330054/2023|2023-04-28|PB1|EPI_ISL_18900596|PB1
A/chicken/South_Korea/2330055/2023|2023-04-28|PB1|EPI_ISL_18900597|PB1
A/pigeon/South_Korea/N23-036/2023|2023-03-30|PB1|EPI_ISL_18900578|PB1
A/chicken/South_Korea/2330053/2023|2023-04-25|PB1|EPI_ISL_18900595|PB1
A/chicken/South_Korea/2330056/2023|2023-04-28|PB1|EPI_ISL_18900598|PB1
A/chicken/South_Korea/2330076/2023|2023-06-22|PB1|EPI_ISL_18900599|PB1
A/chicken/South_Korea/2330046/2023|2023-04-18|PB1|EPI_ISL_18900593|PB1
A/chicken/South_Korea/N23-058/2023|2023-05-15|PB1|EPI_ISL_19631523|PB1
A/chicken/South_Korea/N22-62/2022|2022-06-22|PB1|EPI_ISL_19631516|PB1
A/chicken/South_Korea/N22-142/2022|2022-09-01|PB1|EPI_ISL_19631509|PB1
A/duck/South_Korea/N22-121/2022|2022-09-01|PB1|EPI_ISL_19631524|PB1
A/chicken/South_Korea/N22-143/2022|2022-09-01|PB1|EPI_ISL_19631510|PB1
A/duck/South_Korea/N22-122/2022|2022-09-01|PB1|EPI_ISL_19631525|PB1
A/chicken/South_Korea/N22-152/2022|2022-11-10|PB1|EPI_ISL_19631511|PB1
A/chicken/South_Korea/N23-026/2023|2023-03-30|PB1|EPI_ISL_19631520|PB1
A/duck/South_Korea/N22-161/2022|2022-11-10|PB1|EPI_ISL_19631526|PB1
A/chicken/South_Korea/N23-015/2023|2023-01-13|PB1|EPI_ISL_19631519|PB1
A/chicken/South_Korea/N23-010/2023|2023-01-13|PB1|EPI_ISL_19631518|PB1

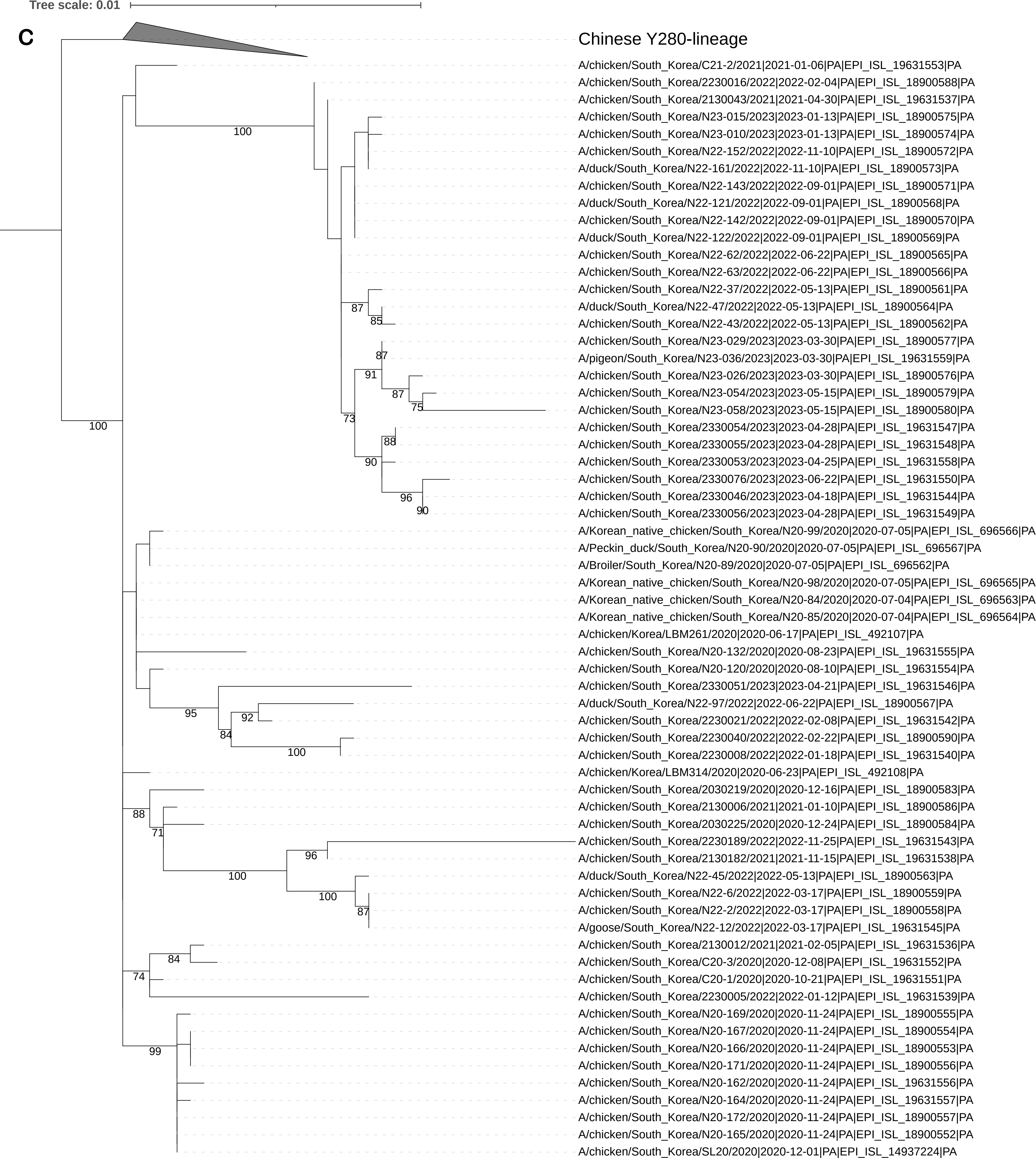
Tree scale: 0.01
C
Chinese Y280-lineage
A/chicken/South_Korea/C21-2/2021|2021-01-06|PA|EPI_ISL_19631553|PA
A/chicken/South_Korea/2230016/2022|2022-02-04|PA|EPI_ISL_18900588|PA
A/chicken/South_Korea/2130043/2021|2021-04-30|PA|EPI_ISL_19631537|PA
A/chicken/South_Korea/N23-015/2023|2023-01-13|PA|EPI_ISL_18900575|PA
A/chicken/South_Korea/N23-010/2023|2023-01-13|PA|EPI_ISL_18900574|PA
A/chicken/South_Korea/N22-152/2022|2022-11-10|PA|EPI_ISL_18900572|PA
A/duck/South_Korea/N22-161/2022|2022-11-10|PA|EPI_ISL_18900573|PA
A/chicken/South_Korea/N22-143/2022|2022-09-01|PA|EPI_ISL_18900571|PA
A/duck/South_Korea/N22-121/2022|2022-09-01|PA|EPI_ISL_18900568|PA
A/chicken/South_Korea/N22-142/2022|2022-09-01|PA|EPI_ISL_18900570|PA
A/duck/South_Korea/N22-122/2022|2022-09-01|PA|EPI_ISL_18900569|PA
A/chicken/South_Korea/N22-62/2022|2022-06-22|PA|EPI_ISL_18900565|PA
A/chicken/South_Korea/N22-63/2022|2022-06-22|PA|EPI_ISL_18900566|PA
A/chicken/South_Korea/N22-37/2022|2022-05-13|PA|EPI_ISL_18900561|PA
A/duck/South_Korea/N22-47/2022|2022-05-13|PA|EPI_ISL_18900564|PA
A/chicken/South_Korea/N22-43/2022|2022-05-13|PA|EPI_ISL_18900562|PA
A/chicken/South_Korea/N23-029/2023|2023-03-30|PA|EPI_ISL_18900577|PA
A/pigeon/South_Korea/N23-036/2023|2023-03-30|PA|EPI_ISL_19631559|PA
A/chicken/South_Korea/N23-026/2023|2023-03-30|PA|EPI_ISL_18900576|PA
A/chicken/South_Korea/N23-054/2023|2023-05-15|PA|EPI_ISL_18900579|PA
A/chicken/South_Korea/N23-058/2023|2023-05-15|PA|EPI_ISL_18900580|PA
A/chicken/South_Korea/2330054/2023|2023-04-28|PA|EPI_ISL_19631547|PA
A/chicken/South_Korea/2330055/2023|2023-04-28|PA|EPI_ISL_19631548|PA
A/chicken/South_Korea/2330053/2023|2023-04-25|PA|EPI_ISL_19631558|PA
A/chicken/South_Korea/2330076/2023|2023-06-22|PA|EPI_ISL_19631550|PA
A/chicken/South_Korea/2330046/2023|2023-04-18|PA|EPI_ISL_19631544|PA
A/chicken/South_Korea/2330056/2023|2023-04-28|PA|EPI_ISL_19631549|PA
A/Korean_native_chicken/South_Korea/N20-99/2020|2020-07-05|PA|EPI_ISL_696566|PA
A/Peckin_duck/South_Korea/N20-90/2020|2020-07-05|PA|EPI_ISL_696567|PA
A/Broiler/South_Korea/N20-89/2020|2020-07-05|PA|EPI_ISL_696562|PA
A/Korean_native_chicken/South_Korea/N20-98/2020|2020-07-05|PA|EPI_ISL_696565|PA
A/Korean_native_chicken/South_Korea/N20-84/2020|2020-07-04|PA|EPI_ISL_696563|PA
A/Korean_native_chicken/South_Korea/N20-85/2020|2020-07-04|PA|EPI_ISL_696564|PA
A/chicken/Korea/LBM261/2020|2020-06-17|PA|EPI_ISL_492107|PA
A/chicken/South_Korea/N20-132/2020|2020-08-23|PA|EPI_ISL_19631555|PA
A/chicken/South_Korea/N20-120/2020|2020-08-10|PA|EPI_ISL_19631554|PA
A/chicken/South_Korea/2330051/2023|2023-04-21|PA|EPI_ISL_19631546|PA
A/duck/South_Korea/N22-97/2022|2022-06-22|PA|EPI_ISL_18900567|PA
A/chicken/South_Korea/2230021/2022|2022-02-08|PA|EPI_ISL_19631542|PA
A/chicken/South_Korea/2230040/2022|2022-02-22|PA|EPI_ISL_18900590|PA
A/chicken/South_Korea/2230008/2022|2022-01-18|PA|EPI_ISL_19631540|PA
A/chicken/Korea/LBM314/2020|2020-06-23|PA|EPI_ISL_492108|PA
A/chicken/South_Korea/2030219/2020|2020-12-16|PA|EPI_ISL_18900583|PA
A/chicken/South_Korea/2130006/2021|2021-01-10|PA|EPI_ISL_18900586|PA
A/chicken/South_Korea/2030225/2020|2020-12-24|PA|EPI_ISL_18900584|PA
A/chicken/South_Korea/2230189/2022|2022-11-25|PA|EPI_ISL_19631543|PA
A/chicken/South_Korea/2130182/2021|2021-11-15|PA|EPI_ISL_19631538|PA
A/duck/South_Korea/N22-45/2022|2022-05-13|PA|EPI_ISL_18900563|PA
A/chicken/South_Korea/N22-6/2022|2022-03-17|PA|EPI_ISL_18900559|PA
A/chicken/South_Korea/N22-2/2022|2022-03-17|PA|EPI_ISL_18900558|PA
A/goose/South_Korea/N22-12/2022|2022-03-17|PA|EPI_ISL_19631545|PA
A/chicken/South_Korea/2130012/2021|2021-02-05|PA|EPI_ISL_19631536|PA
A/chicken/South_Korea/C20-3/2020|2020-12-08|PA|EPI_ISL_19631552|PA
A/chicken/South_Korea/C20-1/2020|2020-10-21|PA|EPI_ISL_19631551|PA
A/chicken/South_Korea/2230005/2022|2022-01-12|PA|EPI_ISL_19631539|PA
A/chicken/South_Korea/N20-169/2020|2020-11-24|PA|EPI_ISL_18900555|PA
A/chicken/South_Korea/N20-167/2020|2020-11-24|PA|EPI_ISL_18900554|PA
A/chicken/South_Korea/N20-166/2020|2020-11-24|PA|EPI_ISL_18900553|PA
A/chicken/South_Korea/N20-171/2020|2020-11-24|PA|EPI_ISL_18900556|PA
A/chicken/South_Korea/N20-162/2020|2020-11-24|PA|EPI_ISL_19631556|PA
A/chicken/South_Korea/N20-164/2020|2020-11-24|PA|EPI_ISL_19631557|PA
A/chicken/South_Korea/N20-172/2020|2020-11-24|PA|EPI_ISL_18900557|PA
A/chicken/South_Korea/N20-165/2020|2020-11-24|PA|EPI_ISL_18900552|PA
A/chicken/South_Korea/SL20/2020|2020-12-01|PA|EPI_ISL_14937224|PA

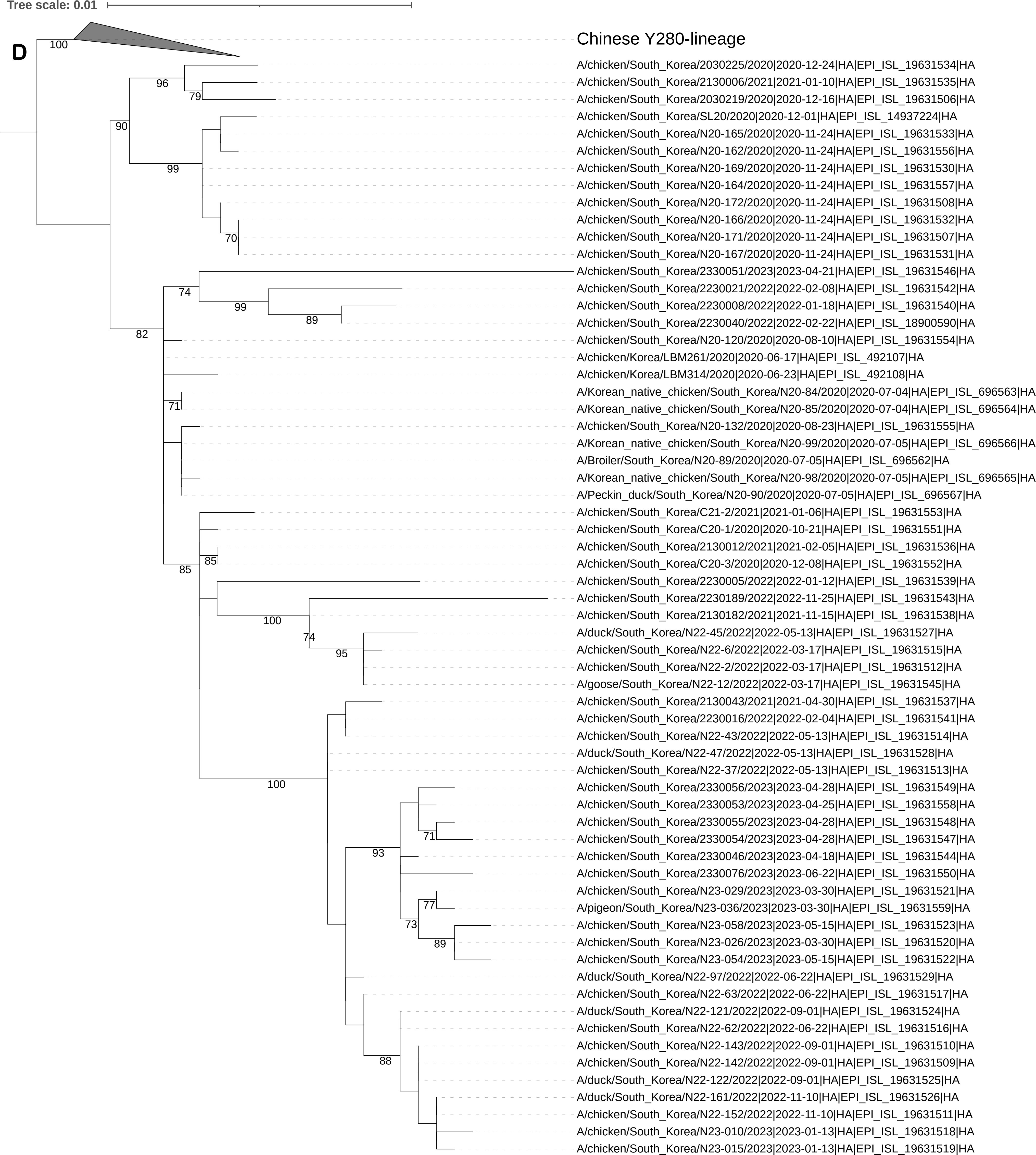
Tree scale: 0.01
D
Chinese Y280-lineage
A/chicken/South_Korea/2030225/2020|2020-12-24|HA|EPI_ISL_19631534|HA
A/chicken/South_Korea/2130006/2021|2021-01-10|HA|EPI_ISL_19631535|HA
A/chicken/South_Korea/2030219/2020|2020-12-16|HA|EPI_ISL_19631506|HA
A/chicken/South_Korea/SL20/2020|2020-12-01|HA|EPI_ISL_14937224|HA
A/chicken/South_Korea/N20-165/2020|2020-11-24|HA|EPI_ISL_19631533|HA
A/chicken/South_Korea/N20-162/2020|2020-11-24|HA|EPI_ISL_19631556|HA
A/chicken/South_Korea/N20-169/2020|2020-11-24|HA|EPI_ISL_19631530|HA
A/chicken/South_Korea/N20-164/2020|2020-11-24|HA|EPI_ISL_19631557|HA
A/chicken/South_Korea/N20-172/2020|2020-11-24|HA|EPI_ISL_19631508|HA
A/chicken/South_Korea/N20-166/2020|2020-11-24|HA|EPI_ISL_19631532|HA
A/chicken/South_Korea/N20-171/2020|2020-11-24|HA|EPI_ISL_19631507|HA
A/chicken/South_Korea/N20-167/2020|2020-11-24|HA|EPI_ISL_19631531|HA
A/chicken/South_Korea/2330051/2023|2023-04-21|HA|EPI_ISL_19631546|HA
A/chicken/South_Korea/2230021/2022|2022-02-08|HA|EPI_ISL_19631542|HA
A/chicken/South_Korea/2230008/2022|2022-01-18|HA|EPI_ISL_19631540|HA
A/chicken/South_Korea/2230040/2022|2022-02-22|HA|EPI_ISL_18900590|HA
A/chicken/South_Korea/N20-120/2020|2020-08-10|HA|EPI_ISL_19631554|HA
A/chicken/Korea/LBM261/2020|2020-06-17|HA|EPI_ISL_492107|HA
A/chicken/Korea/LBM314/2020|2020-06-23|HA|EPI_ISL_492108|HA
A/Korean_native_chicken/South_Korea/N20-84/2020|2020-07-04|HA|EPI_ISL_696563|HA
A/Korean_native_chicken/South_Korea/N20-85/2020|2020-07-04|HA|EPI_ISL_696564|HA
A/chicken/South_Korea/N20-132/2020|2020-08-23|HA|EPI_ISL_19631555|HA
A/Korean_native_chicken/South_Korea/N20-99/2020|2020-07-05|HA|EPI_ISL_696566|HA
A/Broiler/South_Korea/N20-89/2020|2020-07-05|HA|EPI_ISL_696562|HA
A/Korean_native_chicken/South_Korea/N20-98/2020|2020-07-05|HA|EPI_ISL_696565|HA
A/Peckin_duck/South_Korea/N20-90/2020|2020-07-05|HA|EPI_ISL_696567|HA
A/chicken/South_Korea/C21-2/2021|2021-01-06|HA|EPI_ISL_19631553|HA
A/chicken/South_Korea/C20-1/2020|2020-10-21|HA|EPI_ISL_19631551|HA
A/chicken/South_Korea/2130012/2021|2021-02-05|HA|EPI_ISL_19631536|HA
A/chicken/South_Korea/C20-3/2020|2020-12-08|HA|EPI_ISL_19631552|HA
A/chicken/South_Korea/2230005/2022|2022-01-12|HA|EPI_ISL_19631539|HA
A/chicken/South_Korea/2230189/2022|2022-11-25|HA|EPI_ISL_19631543|HA
A/chicken/South_Korea/2130182/2021|2021-11-15|HA|EPI_ISL_19631538|HA
A/duck/South_Korea/N22-45/2022|2022-05-13|HA|EPI_ISL_19631527|HA
A/chicken/South_Korea/N22-6/2022|2022-03-17|HA|EPI_ISL_19631515|HA
A/chicken/South_Korea/N22-2/2022|2022-03-17|HA|EPI_ISL_19631512|HA
A/goose/South_Korea/N22-12/2022|2022-03-17|HA|EPI_ISL_19631545|HA
A/chicken/South_Korea/2130043/2021|2021-04-30|HA|EPI_ISL_19631537|HA
A/chicken/South_Korea/2230016/2022|2022-02-04|HA|EPI_ISL_19631541|HA
A/chicken/South_Korea/N22-43/2022|2022-05-13|HA|EPI_ISL_19631514|HA
A/duck/South_Korea/N22-47/2022|2022-05-13|HA|EPI_ISL_19631528|HA
A/chicken/South_Korea/N22-37/2022|2022-05-13|HA|EPI_ISL_19631513|HA
A/chicken/South_Korea/2330056/2023|2023-04-28|HA|EPI_ISL_19631549|HA
A/chicken/South_Korea/2330053/2023|2023-04-25|HA|EPI_ISL_19631558|HA
A/chicken/South_Korea/2330055/2023|2023-04-28|HA|EPI_ISL_19631548|HA
A/chicken/South_Korea/2330054/2023|2023-04-28|HA|EPI_ISL_19631547|HA
A/chicken/South_Korea/2330046/2023|2023-04-18|HA|EPI_ISL_19631544|HA
A/chicken/South_Korea/2330076/2023|2023-06-22|HA|EPI_ISL_19631550|HA
A/chicken/South_Korea/N23-029/2023|2023-03-30|HA|EPI_ISL_19631521|HA
A/pigeon/South_Korea/N23-036/2023|2023-03-30|HA|EPI_ISL_19631559|HA
A/chicken/South_Korea/N23-058/2023|2023-05-15|HA|EPI_ISL_19631523|HA
A/chicken/South_Korea/N23-026/2023|2023-03-30|HA|EPI_ISL_19631520|HA
A/chicken/South_Korea/N23-054/2023|2023-05-15|HA|EPI_ISL_19631522|HA
A/duck/South_Korea/N22-97/2022|2022-06-22|HA|EPI_ISL_19631529|HA
A/chicken/South_Korea/N22-63/2022|2022-06-22|HA|EPI_ISL_19631517|HA
A/duck/South_Korea/N22-121/2022|2022-09-01|HA|EPI_ISL_19631524|HA
A/chicken/South_Korea/N22-62/2022|2022-06-22|HA|EPI_ISL_19631516|HA
A/chicken/South_Korea/N22-143/2022|2022-09-01|HA|EPI_ISL_19631510|HA
A/chicken/South_Korea/N22-142/2022|2022-09-01|HA|EPI_ISL_19631509|HA
A/duck/South_Korea/N22-122/2022|2022-09-01|HA|EPI_ISL_19631525|HA
A/duck/South_Korea/N22-161/2022|2022-11-10|HA|EPI_ISL_19631526|HA
A/chicken/South_Korea/N22-152/2022|2022-11-10|HA|EPI_ISL_19631511|HA
A/chicken/South_Korea/N23-010/2023|2023-01-13|HA|EPI_ISL_19631518|HA
A/chicken/South_Korea/N23-015/2023|2023-01-13|HA|EPI_ISL_19631519|HA

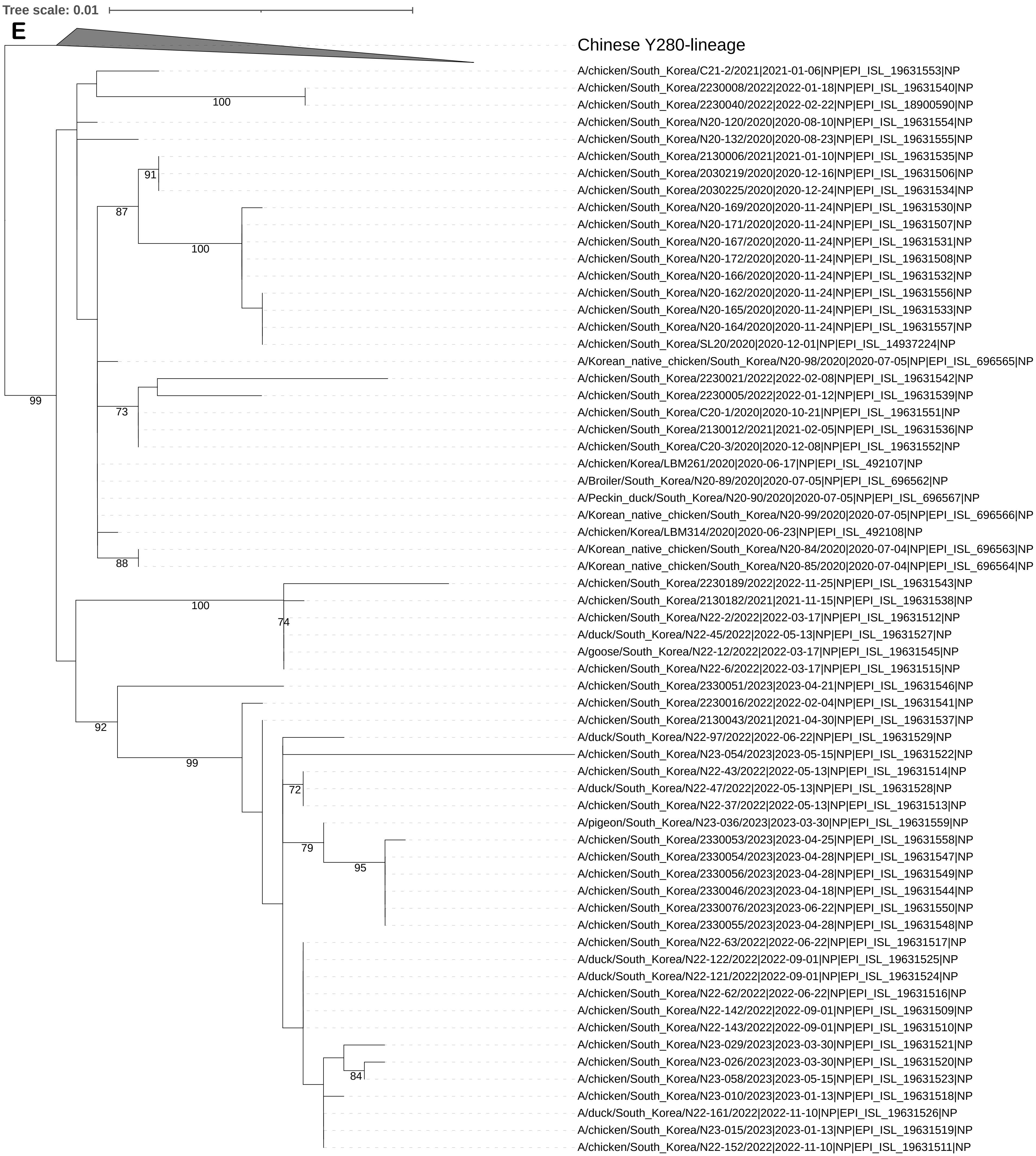
Tree scale: 0.01
E
Chinese Y280-lineage
A/chicken/South_Korea/C21-2/2021|2021-01-06|NP|EPI_ISL_19631553|NP
A/chicken/South_Korea/2230008/2022|2022-01-18|NP|EPI_ISL_19631540|NP
A/chicken/South_Korea/2230040/2022|2022-02-22|NP|EPI_ISL_18900590|NP
A/chicken/South_Korea/N20-120/2020|2020-08-10|NP|EPI_ISL_19631554|NP
A/chicken/South_Korea/N20-132/2020|2020-08-23|NP|EPI_ISL_19631555|NP
A/chicken/South_Korea/2130006/2021|2021-01-10|NP|EPI_ISL_19631535|NP
A/chicken/South_Korea/2030219/2020|2020-12-16|NP|EPI_ISL_19631506|NP
A/chicken/South_Korea/2030225/2020|2020-12-24|NP|EPI_ISL_19631534|NP
A/chicken/South_Korea/N20-169/2020|2020-11-24|NP|EPI_ISL_19631530|NP
A/chicken/South_Korea/N20-171/2020|2020-11-24|NP|EPI_ISL_19631507|NP
A/chicken/South_Korea/N20-167/2020|2020-11-24|NP|EPI_ISL_19631531|NP
A/chicken/South_Korea/N20-172/2020|2020-11-24|NP|EPI_ISL_19631508|NP
A/chicken/South_Korea/N20-166/2020|2020-11-24|NP|EPI_ISL_19631532|NP
A/chicken/South_Korea/N20-162/2020|2020-11-24|NP|EPI_ISL_19631556|NP
A/chicken/South_Korea/N20-165/2020|2020-11-24|NP|EPI_ISL_19631533|NP
A/chicken/South_Korea/N20-164/2020|2020-11-24|NP|EPI_ISL_19631557|NP
A/chicken/South_Korea/SL20/2020|2020-12-01|NP|EPI_ISL_14937224|NP
A/Korean_native_chicken/South_Korea/N20-98/2020|2020-07-05|NP|EPI_ISL_696565|NP
A/chicken/South_Korea/2230021/2022|2022-02-08|NP|EPI_ISL_19631542|NP
A/chicken/South_Korea/2230005/2022|2022-01-12|NP|EPI_ISL_19631539|NP
A/chicken/South_Korea/C20-1/2020|2020-10-21|NP|EPI_ISL_19631551|NP
A/chicken/South_Korea/2130012/2021|2021-02-05|NP|EPI_ISL_19631536|NP
A/chicken/South_Korea/C20-3/2020|2020-12-08|NP|EPI_ISL_19631552|NP
A/chicken/Korea/LBM261/2020|2020-06-17|NP|EPI_ISL_492107|NP
A/Broiler/South_Korea/N20-89/2020|2020-07-05|NP|EPI_ISL_696562|NP
A/Peckin_duck/South_Korea/N20-90/2020|2020-07-05|NP|EPI_ISL_696567|NP
A/Korean_native_chicken/South_Korea/N20-99/2020|2020-07-05|NP|EPI_ISL_696566|NP
A/chicken/Korea/LBM314/2020|2020-06-23|NP|EPI_ISL_492108|NP
A/Korean_native_chicken/South_Korea/N20-84/2020|2020-07-04|NP|EPI_ISL_696563|NP
A/Korean_native_chicken/South_Korea/N20-85/2020|2020-07-04|NP|EPI_ISL_696564|NP
A/chicken/South_Korea/2230189/2022|2022-11-25|NP|EPI_ISL_19631543|NP
A/chicken/South_Korea/2130182/2021|2021-11-15|NP|EPI_ISL_19631538|NP
A/chicken/South_Korea/N22-2/2022|2022-03-17|NP|EPI_ISL_19631512|NP
A/duck/South_Korea/N22-45/2022|2022-05-13|NP|EPI_ISL_19631527|NP
A/goose/South_Korea/N22-12/2022|2022-03-17|NP|EPI_ISL_19631545|NP
A/chicken/South_Korea/N22-6/2022|2022-03-17|NP|EPI_ISL_19631515|NP
A/chicken/South_Korea/2330051/2023|2023-04-21|NP|EPI_ISL_19631546|NP
A/chicken/South_Korea/2230016/2022|2022-02-04|NP|EPI_ISL_19631541|NP
A/chicken/South_Korea/2130043/2021|2021-04-30|NP|EPI_ISL_19631537|NP
A/duck/South_Korea/N22-97/2022|2022-06-22|NP|EPI_ISL_19631529|NP
A/chicken/South_Korea/N23-054/2023|2023-05-15|NP|EPI_ISL_19631522|NP
A/chicken/South_Korea/N22-43/2022|2022-05-13|NP|EPI_ISL_19631514|NP
A/duck/South_Korea/N22-47/2022|2022-05-13|NP|EPI_ISL_19631528|NP
A/chicken/South_Korea/N22-37/2022|2022-05-13|NP|EPI_ISL_19631513|NP
A/pigeon/South_Korea/N23-036/2023|2023-03-30|NP|EPI_ISL_19631559|NP
A/chicken/South_Korea/2330053/2023|2023-04-25|NP|EPI_ISL_19631558|NP
A/chicken/South_Korea/2330054/2023|2023-04-28|NP|EPI_ISL_19631547|NP
A/chicken/South_Korea/2330056/2023|2023-04-28|NP|EPI_ISL_19631549|NP
A/chicken/South_Korea/2330046/2023|2023-04-18|NP|EPI_ISL_19631544|NP
A/chicken/South_Korea/2330076/2023|2023-06-22|NP|EPI_ISL_19631550|NP
A/chicken/South_Korea/2330055/2023|2023-04-28|NP|EPI_ISL_19631548|NP
A/chicken/South_Korea/N22-63/2022|2022-06-22|NP|EPI_ISL_19631517|NP
A/duck/South_Korea/N22-122/2022|2022-09-01|NP|EPI_ISL_19631525|NP
A/duck/South_Korea/N22-121/2022|2022-09-01|NP|EPI_ISL_19631524|NP
A/chicken/South_Korea/N22-62/2022|2022-06-22|NP|EPI_ISL_19631516|NP
A/chicken/South_Korea/N22-142/2022|2022-09-01|NP|EPI_ISL_19631509|NP
A/chicken/South_Korea/N22-143/2022|2022-09-01|NP|EPI_ISL_19631510|NP
A/chicken/South_Korea/N23-029/2023|2023-03-30|NP|EPI_ISL_19631521|NP
A/chicken/South_Korea/N23-026/2023|2023-03-30|NP|EPI_ISL_19631520|NP
A/chicken/South_Korea/N23-058/2023|2023-05-15|NP|EPI_ISL_19631523|NP
A/chicken/South_Korea/N23-010/2023|2023-01-13|NP|EPI_ISL_19631518|NP
A/duck/South_Korea/N22-161/2022|2022-11-10|NP|EPI_ISL_19631526|NP
A/chicken/South_Korea/N23-015/2023|2023-01-13|NP|EPI_ISL_19631519|NP
A/chicken/South_Korea/N22-152/2022|2022-11-10|NP|EPI_ISL_19631511|NP
100
91
87
100
99
73
88
100
74
92
99
72
79
95
84

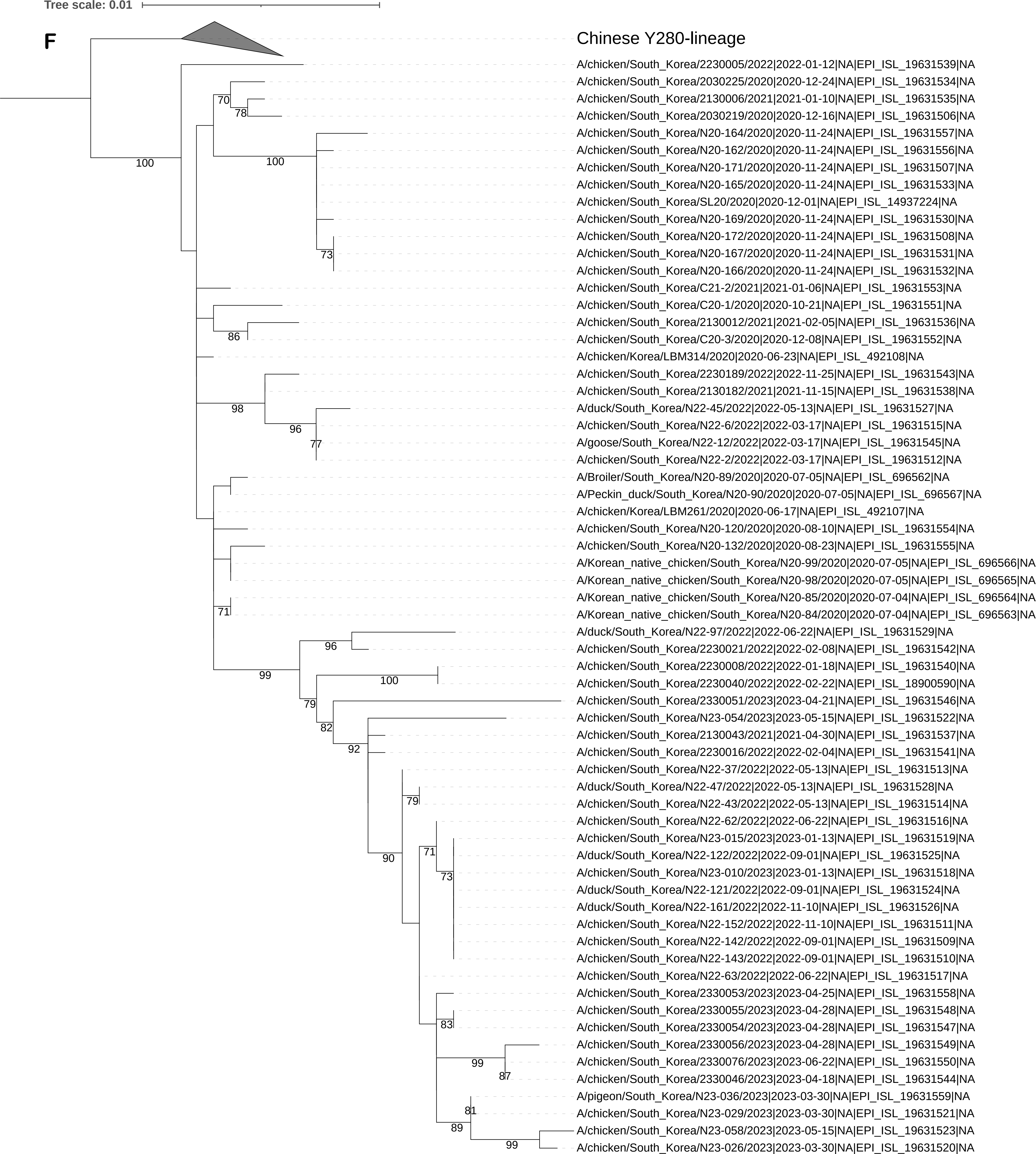

Tree scale: 0.01
F
Chinese Y280-lineage
A/chicken/South_Korea/2230005/2022|2022-01-12|NA|EPI_ISL_19631539|NA
A/chicken/South_Korea/2030225/2020|2020-12-24|NA|EPI_ISL_19631534|NA
A/chicken/South_Korea/2130006/2021|2021-01-10|NA|EPI_ISL_19631535|NA
A/chicken/South_Korea/2030219/2020|2020-12-16|NA|EPI_ISL_19631506|NA
A/chicken/South_Korea/N20-164/2020|2020-11-24|NA|EPI_ISL_19631557|NA
A/chicken/South_Korea/N20-162/2020|2020-11-24|NA|EPI_ISL_19631556|NA
A/chicken/South_Korea/N20-171/2020|2020-11-24|NA|EPI_ISL_19631507|NA
A/chicken/South_Korea/N20-165/2020|2020-11-24|NA|EPI_ISL_19631533|NA
A/chicken/South_Korea/SL20/2020|2020-12-01|NA|EPI_ISL_14937224|NA
A/chicken/South_Korea/N20-169/2020|2020-11-24|NA|EPI_ISL_19631530|NA
A/chicken/South_Korea/N20-172/2020|2020-11-24|NA|EPI_ISL_19631508|NA
A/chicken/South_Korea/N20-167/2020|2020-11-24|NA|EPI_ISL_19631531|NA
A/chicken/South_Korea/N20-166/2020|2020-11-24|NA|EPI_ISL_19631532|NA
A/chicken/South_Korea/C21-2/2021|2021-01-06|NA|EPI_ISL_19631553|NA
A/chicken/South_Korea/C20-1/2020|2020-10-21|NA|EPI_ISL_19631551|NA
A/chicken/South_Korea/2130012/2021|2021-02-05|NA|EPI_ISL_19631536|NA
A/chicken/South_Korea/C20-3/2020|2020-12-08|NA|EPI_ISL_19631552|NA
A/chicken/Korea/LBM314/2020|2020-06-23|NA|EPI_ISL_492108|NA
A/chicken/South_Korea/2230189/2022|2022-11-25|NA|EPI_ISL_19631543|NA
A/chicken/South_Korea/2130182/2021|2021-11-15|NA|EPI_ISL_19631538|NA
A/duck/South_Korea/N22-45/2022|2022-05-13|NA|EPI_ISL_19631527|NA
A/chicken/South_Korea/N22-6/2022|2022-03-17|NA|EPI_ISL_19631515|NA
A/goose/South_Korea/N22-12/2022|2022-03-17|NA|EPI_ISL_19631545|NA
A/chicken/South_Korea/N22-2/2022|2022-03-17|NA|EPI_ISL_19631512|NA
A/Broiler/South_Korea/N20-89/2020|2020-07-05|NA|EPI_ISL_696562|NA
A/Peckin_duck/South_Korea/N20-90/2020|2020-07-05|NA|EPI_ISL_696567|NA
A/chicken/Korea/LBM261/2020|2020-06-17|NA|EPI_ISL_492107|NA
A/chicken/South_Korea/N20-120/2020|2020-08-10|NA|EPI_ISL_19631554|NA
A/chicken/South_Korea/N20-132/2020|2020-08-23|NA|EPI_ISL_19631555|NA
A/Korean_native_chicken/South_Korea/N20-99/2020|2020-07-05|NA|EPI_ISL_696566|NA
A/Korean_native_chicken/South_Korea/N20-98/2020|2020-07-05|NA|EPI_ISL_696565|NA
A/Korean_native_chicken/South_Korea/N20-85/2020|2020-07-04|NA|EPI_ISL_696564|NA
A/Korean_native_chicken/South_Korea/N20-84/2020|2020-07-04|NA|EPI_ISL_696563|NA
A/duck/South_Korea/N22-97/2022|2022-06-22|NA|EPI_ISL_19631529|NA
A/chicken/South_Korea/2230021/2022|2022-02-08|NA|EPI_ISL_19631542|NA
A/chicken/South_Korea/2230008/2022|2022-01-18|NA|EPI_ISL_19631540|NA
A/chicken/South_Korea/2230040/2022|2022-02-22|NA|EPI_ISL_18900590|NA
A/chicken/South_Korea/2330051/2023|2023-04-21|NA|EPI_ISL_19631546|NA
A/chicken/South_Korea/N23-054/2023|2023-05-15|NA|EPI_ISL_19631522|NA
A/chicken/South_Korea/2130043/2021|2021-04-30|NA|EPI_ISL_19631537|NA
A/chicken/South_Korea/2230016/2022|2022-02-04|NA|EPI_ISL_19631541|NA
A/chicken/South_Korea/N22-37/2022|2022-05-13|NA|EPI_ISL_19631513|NA
A/duck/South_Korea/N22-47/2022|2022-05-13|NA|EPI_ISL_19631528|NA
A/chicken/South_Korea/N22-43/2022|2022-05-13|NA|EPI_ISL_19631514|NA
A/chicken/South_Korea/N22-62/2022|2022-06-22|NA|EPI_ISL_19631516|NA
A/chicken/South_Korea/N23-015/2023|2023-01-13|NA|EPI_ISL_19631519|NA
A/duck/South_Korea/N22-122/2022|2022-09-01|NA|EPI_ISL_19631525|NA
A/chicken/South_Korea/N23-010/2023|2023-01-13|NA|EPI_ISL_19631518|NA
A/duck/South_Korea/N22-121/2022|2022-09-01|NA|EPI_ISL_19631524|NA
A/duck/South_Korea/N22-161/2022|2022-11-10|NA|EPI_ISL_19631526|NA
A/chicken/South_Korea/N22-152/2022|2022-11-10|NA|EPI_ISL_19631511|NA
A/chicken/South_Korea/N22-142/2022|2022-09-01|NA|EPI_ISL_19631509|NA
A/chicken/South_Korea/N22-143/2022|2022-09-01|NA|EPI_ISL_19631510|NA
A/chicken/South_Korea/N22-63/2022|2022-06-22|NA|EPI_ISL_19631517|NA
A/chicken/South_Korea/2330053/2023|2023-04-25|NA|EPI_ISL_19631558|NA
A/chicken/South_Korea/2330055/2023|2023-04-28|NA|EPI_ISL_19631548|NA
A/chicken/South_Korea/2330054/2023|2023-04-28|NA|EPI_ISL_19631547|NA
A/chicken/South_Korea/2330056/2023|2023-04-28|NA|EPI_ISL_19631549|NA
A/chicken/South_Korea/2330076/2023|2023-06-22|NA|EPI_ISL_19631550|NA
A/chicken/South_Korea/2330046/2023|2023-04-18|NA|EPI_ISL_19631544|NA
A/pigeon/South_Korea/N23-036/2023|2023-03-30|NA|EPI_ISL_19631559|NA
A/chicken/South_Korea/N23-029/2023|2023-03-30|NA|EPI_ISL_19631521|NA
A/chicken/South_Korea/N23-058/2023|2023-05-15|NA|EPI_ISL_19631523|NA
A/chicken/South_Korea/N23-026/2023|2023-03-30|NA|EPI_ISL_19631520|NA

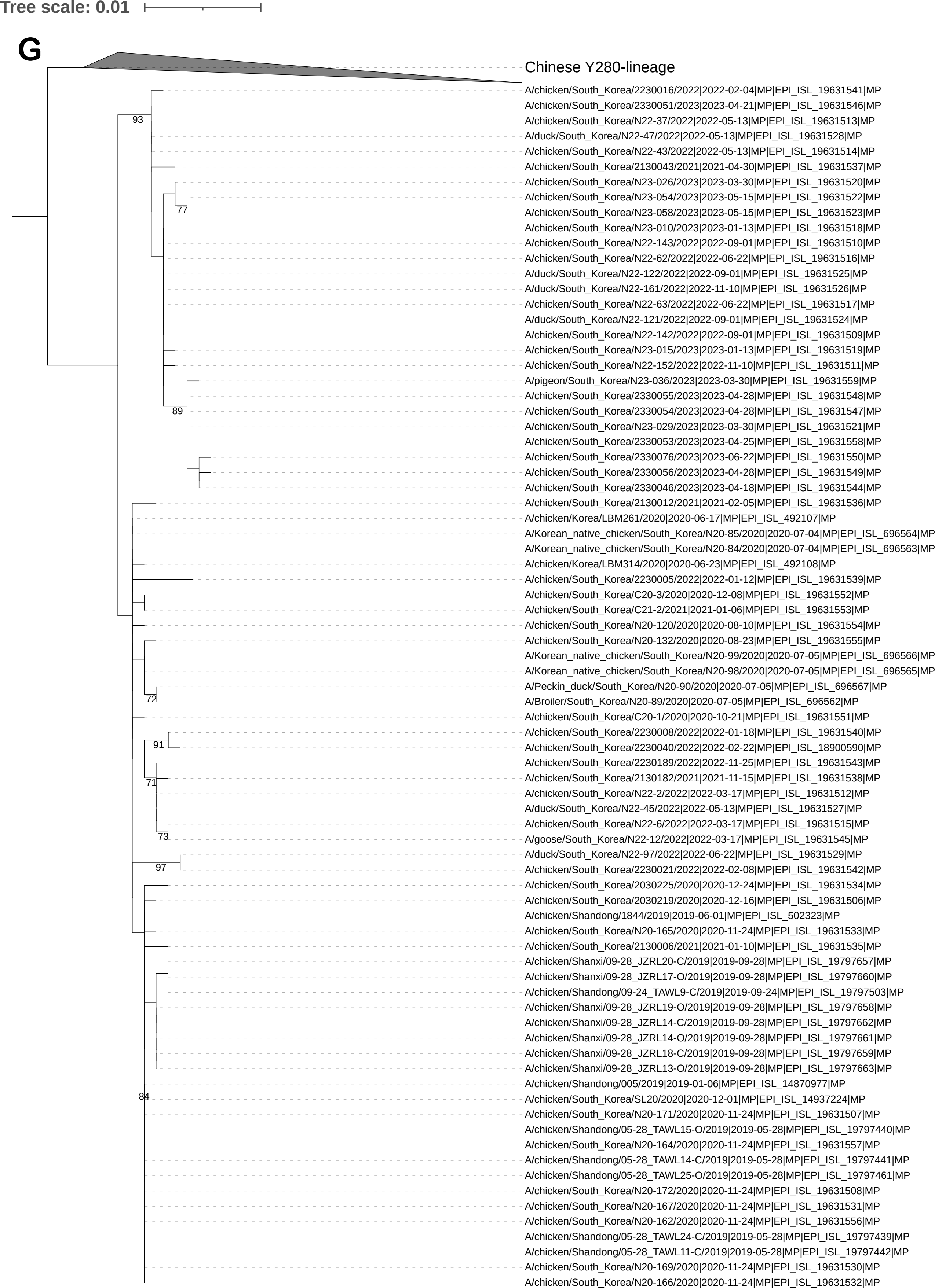

Tree scale: 0.01
G
93
77
89
72
91
71
73
97
84
Chinese Y280-lineage
A/chicken/South_Korea/2230016/2022|2022-02-04|MP|EPI_ISL_19631541|MP
A/chicken/South_Korea/2330051/2023|2023-04-21|MP|EPI_ISL_19631546|MP
A/chicken/South_Korea/N22-37/2022|2022-05-13|MP|EPI_ISL_19631513|MP
A/duck/South_Korea/N22-47/2022|2022-05-13|MP|EPI_ISL_19631528|MP
A/chicken/South_Korea/N22-43/2022|2022-05-13|MP|EPI_ISL_19631514|MP
A/chicken/South_Korea/2130043/2021|2021-04-30|MP|EPI_ISL_19631537|MP
A/chicken/South_Korea/N23-026/2023|2023-03-30|MP|EPI_ISL_19631520|MP
A/chicken/South_Korea/N23-054/2023|2023-05-15|MP|EPI_ISL_19631522|MP
A/chicken/South_Korea/N23-058/2023|2023-05-15|MP|EPI_ISL_19631523|MP
A/chicken/South_Korea/N23-010/2023|2023-01-13|MP|EPI_ISL_19631518|MP
A/chicken/South_Korea/N22-143/2022|2022-09-01|MP|EPI_ISL_19631510|MP
A/chicken/South_Korea/N22-62/2022|2022-06-22|MP|EPI_ISL_19631516|MP
A/duck/South_Korea/N22-122/2022|2022-09-01|MP|EPI_ISL_19631525|MP
A/duck/South_Korea/N22-161/2022|2022-11-10|MP|EPI_ISL_19631526|MP
A/chicken/South_Korea/N22-63/2022|2022-06-22|MP|EPI_ISL_19631517|MP
A/duck/South_Korea/N22-121/2022|2022-09-01|MP|EPI_ISL_19631524|MP
A/chicken/South_Korea/N22-142/2022|2022-09-01|MP|EPI_ISL_19631509|MP
A/chicken/South_Korea/N23-015/2023|2023-01-13|MP|EPI_ISL_19631519|MP
A/chicken/South_Korea/N22-152/2022|2022-11-10|MP|EPI_ISL_19631511|MP
A/pigeon/South_Korea/N23-036/2023|2023-03-30|MP|EPI_ISL_19631559|MP
A/chicken/South_Korea/2330055/2023|2023-04-28|MP|EPI_ISL_19631548|MP
A/chicken/South_Korea/2330054/2023|2023-04-28|MP|EPI_ISL_19631547|MP
A/chicken/South_Korea/N23-029/2023|2023-03-30|MP|EPI_ISL_19631521|MP
A/chicken/South_Korea/2330053/2023|2023-04-25|MP|EPI_ISL_19631558|MP
A/chicken/South_Korea/2330076/2023|2023-06-22|MP|EPI_ISL_19631550|MP
A/chicken/South_Korea/2330056/2023|2023-04-28|MP|EPI_ISL_19631549|MP
A/chicken/South_Korea/2330046/2023|2023-04-18|MP|EPI_ISL_19631544|MP
A/chicken/South_Korea/2130012/2021|2021-02-05|MP|EPI_ISL_19631536|MP
A/chicken/Korea/LBM261/2020|2020-06-17|MP|EPI_ISL_492107|MP
A/Korean_native_chicken/South_Korea/N20-85/2020|2020-07-04|MP|EPI_ISL_696564|MP
A/Korean_native_chicken/South_Korea/N20-84/2020|2020-07-04|MP|EPI_ISL_696563|MP
A/chicken/Korea/LBM314/2020|2020-06-23|MP|EPI_ISL_492108|MP
A/chicken/South_Korea/2230005/2022|2022-01-12|MP|EPI_ISL_19631539|MP
A/chicken/South_Korea/C20-3/2020|2020-12-08|MP|EPI_ISL_19631552|MP
A/chicken/South_Korea/C21-2/2021|2021-01-06|MP|EPI_ISL_19631553|MP
A/chicken/South_Korea/N20-120/2020|2020-08-10|MP|EPI_ISL_19631554|MP
A/chicken/South_Korea/N20-132/2020|2020-08-23|MP|EPI_ISL_19631555|MP
A/Korean_native_chicken/South_Korea/N20-99/2020|2020-07-05|MP|EPI_ISL_696566|MP
A/Korean_native_chicken/South_Korea/N20-98/2020|2020-07-05|MP|EPI_ISL_696565|MP
A/Peckin_duck/South_Korea/N20-90/2020|2020-07-05|MP|EPI_ISL_696567|MP
A/Broiler/South_Korea/N20-89/2020|2020-07-05|MP|EPI_ISL_696562|MP
A/chicken/South_Korea/C20-1/2020|2020-10-21|MP|EPI_ISL_19631551|MP
A/chicken/South_Korea/2230008/2022|2022-01-18|MP|EPI_ISL_19631540|MP
A/chicken/South_Korea/2230040/2022|2022-02-22|MP|EPI_ISL_18900590|MP
A/chicken/South_Korea/2230189/2022|2022-11-25|MP|EPI_ISL_19631543|MP
A/chicken/South_Korea/2130182/2021|2021-11-15|MP|EPI_ISL_19631538|MP
A/chicken/South_Korea/N22-2/2022|2022-03-17|MP|EPI_ISL_19631512|MP
A/duck/South_Korea/N22-45/2022|2022-05-13|MP|EPI_ISL_19631527|MP
A/chicken/South_Korea/N22-6/2022|2022-03-17|MP|EPI_ISL_19631515|MP
A/goose/South_Korea/N22-12/2022|2022-03-17|MP|EPI_ISL_19631545|MP
A/duck/South_Korea/N22-97/2022|2022-06-22|MP|EPI_ISL_19631529|MP
A/chicken/South_Korea/2230021/2022|2022-02-08|MP|EPI_ISL_19631542|MP
A/chicken/South_Korea/2030225/2020|2020-12-24|MP|EPI_ISL_19631534|MP
A/chicken/South_Korea/2030219/2020|2020-12-16|MP|EPI_ISL_19631506|MP
A/chicken/Shandong/1844/2019|2019-06-01|MP|EPI_ISL_502323|MP
A/chicken/South_Korea/N20-165/2020|2020-11-24|MP|EPI_ISL_19631533|MP
A/chicken/South_Korea/2130006/2021|2021-01-10|MP|EPI_ISL_19631535|MP
A/chicken/Shanxi/09-28_JZRL20-C/2019|2019-09-28|MP|EPI_ISL_19797657|MP
A/chicken/Shanxi/09-28_JZRL17-O/2019|2019-09-28|MP|EPI_ISL_19797660|MP
A/chicken/Shandong/09-24_TAWL9-C/2019|2019-09-24|MP|EPI_ISL_19797503|MP
A/chicken/Shanxi/09-28_JZRL19-O/2019|2019-09-28|MP|EPI_ISL_19797658|MP
A/chicken/Shanxi/09-28_JZRL14-C/2019|2019-09-28|MP|EPI_ISL_19797662|MP
A/chicken/Shanxi/09-28_JZRL14-O/2019|2019-09-28|MP|EPI_ISL_19797661|MP
A/chicken/Shanxi/09-28_JZRL18-C/2019|2019-09-28|MP|EPI_ISL_19797659|MP
A/chicken/Shanxi/09-28_JZRL13-O/2019|2019-09-28|MP|EPI_ISL_19797663|MP
A/chicken/Shandong/005/2019|2019-01-06|MP|EPI_ISL_14870977|MP
A/chicken/South_Korea/SL20/2020|2020-12-01|MP|EPI_ISL_14937224|MP
A/chicken/South_Korea/N20-171/2020|2020-11-24|MP|EPI_ISL_19631507|MP
A/chicken/Shandong/05-28_TAWL15-O/2019|2019-05-28|MP|EPI_ISL_19797440|MP
A/chicken/South_Korea/N20-164/2020|2020-11-24|MP|EPI_ISL_19631557|MP
A/chicken/Shandong/05-28_TAWL14-C/2019|2019-05-28|MP|EPI_ISL_19797441|MP
A/chicken/Shandong/05-28_TAWL25-O/2019|2019-05-28|MP|EPI_ISL_19797461|MP
A/chicken/South_Korea/N20-172/2020|2020-11-24|MP|EPI_ISL_19631508|MP
A/chicken/South_Korea/N20-167/2020|2020-11-24|MP|EPI_ISL_19631531|MP
A/chicken/South_Korea/N20-162/2020|2020-11-24|MP|EPI_ISL_19631556|MP
A/chicken/Shandong/05-28_TAWL24-C/2019|2019-05-28|MP|EPI_ISL_19797439|MP
A/chicken/Shandong/05-28_TAWL11-C/2019|2019-05-28|MP|EPI_ISL_19797442|MP
A/chicken/South_Korea/N20-169/2020|2020-11-24|MP|EPI_ISL_19631530|MP
A/chicken/South_Korea/N20-166/2020|2020-11-24|MP|EPI_ISL_19631532|MP

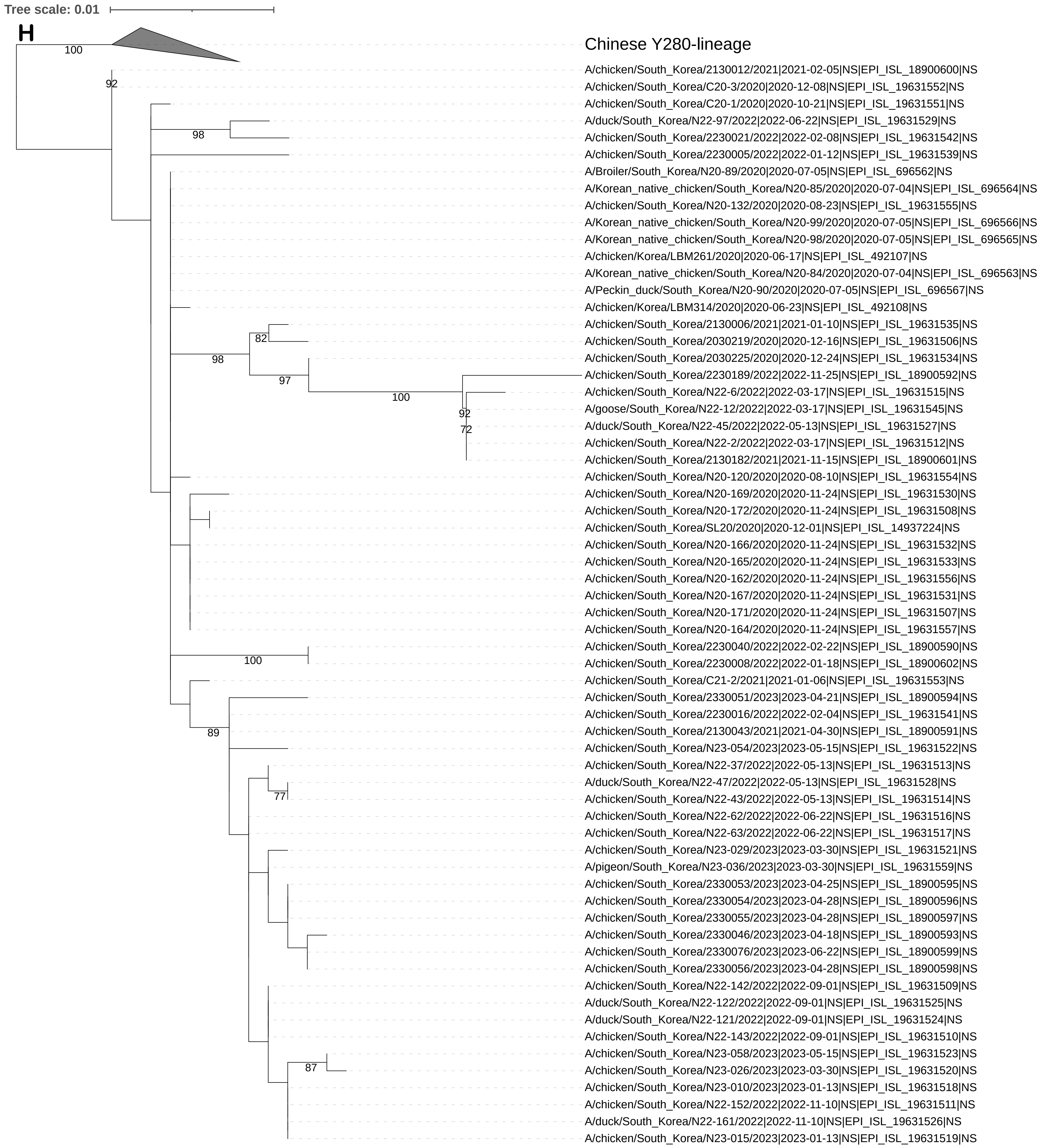

Tree scale: 0.01
H
100
Chinese Y280-lineage
A/chicken/South_Korea/2130012/2021|2021-02-05|NS|EPI_ISL_18900600|NS
92
A/chicken/South_Korea/C20-3/2020|2020-12-08|NS|EPI_ISL_19631552|NS
A/chicken/South_Korea/C20-1/2020|2020-10-21|NS|EPI_ISL_19631551|NS
A/duck/South_Korea/N22-97/2022|2022-06-22|NS|EPI_ISL_19631529|NS
98
A/chicken/South_Korea/2230021/2022|2022-02-08|NS|EPI_ISL_19631542|NS
A/chicken/South_Korea/2230005/2022|2022-01-12|NS|EPI_ISL_19631539|NS
A/Broiler/South_Korea/N20-89/2020|2020-07-05|NS|EPI_ISL_696562|NS
A/Korean_native_chicken/South_Korea/N20-85/2020|2020-07-04|NS|EPI_ISL_696564|NS
A/chicken/South_Korea/N20-132/2020|2020-08-23|NS|EPI_ISL_19631555|NS
A/Korean_native_chicken/South_Korea/N20-99/2020|2020-07-05|NS|EPI_ISL_696566|NS
A/Korean_native_chicken/South_Korea/N20-98/2020|2020-07-05|NS|EPI_ISL_696565|NS
A/chicken/Korea/LBM261/2020|2020-06-17|NS|EPI_ISL_492107|NS
A/Korean_native_chicken/South_Korea/N20-84/2020|2020-07-04|NS|EPI_ISL_696563|NS
A/Peckin_duck/South_Korea/N20-90/2020|2020-07-05|NS|EPI_ISL_696567|NS
A/chicken/Korea/LBM314/2020|2020-06-23|NS|EPI_ISL_492108|NS
A/chicken/South_Korea/2130006/2021|2021-01-10|NS|EPI_ISL_19631535|NS
82
A/chicken/South_Korea/2030219/2020|2020-12-16|NS|EPI_ISL_19631506|NS
98
A/chicken/South_Korea/2030225/2020|2020-12-24|NS|EPI_ISL_19631534|NS
97
A/chicken/South_Korea/2230189/2022|2022-11-25|NS|EPI_ISL_18900592|NS
100
A/chicken/South_Korea/N22-6/2022|2022-03-17|NS|EPI_ISL_19631515|NS
92
A/goose/South_Korea/N22-12/2022|2022-03-17|NS|EPI_ISL_19631545|NS
72
A/duck/South_Korea/N22-45/2022|2022-05-13|NS|EPI_ISL_19631527|NS
A/chicken/South_Korea/N22-2/2022|2022-03-17|NS|EPI_ISL_19631512|NS
A/chicken/South_Korea/2130182/2021|2021-11-15|NS|EPI_ISL_18900601|NS
A/chicken/South_Korea/N20-120/2020|2020-08-10|NS|EPI_ISL_19631554|NS
A/chicken/South_Korea/N20-169/2020|2020-11-24|NS|EPI_ISL_19631530|NS
A/chicken/South_Korea/N20-172/2020|2020-11-24|NS|EPI_ISL_19631508|NS
A/chicken/South_Korea/SL20/2020|2020-12-01|NS|EPI_ISL_14937224|NS
A/chicken/South_Korea/N20-166/2020|2020-11-24|NS|EPI_ISL_19631532|NS
A/chicken/South_Korea/N20-165/2020|2020-11-24|NS|EPI_ISL_19631533|NS
A/chicken/South_Korea/N20-162/2020|2020-11-24|NS|EPI_ISL_19631556|NS
A/chicken/South_Korea/N20-167/2020|2020-11-24|NS|EPI_ISL_19631531|NS
A/chicken/South_Korea/N20-171/2020|2020-11-24|NS|EPI_ISL_19631507|NS
A/chicken/South_Korea/N20-164/2020|2020-11-24|NS|EPI_ISL_19631557|NS
A/chicken/South_Korea/2230040/2022|2022-02-22|NS|EPI_ISL_18900590|NS
100
A/chicken/South_Korea/2230008/2022|2022-01-18|NS|EPI_ISL_18900602|NS
A/chicken/South_Korea/C21-2/2021|2021-01-06|NS|EPI_ISL_19631553|NS
A/chicken/South_Korea/2330051/2023|2023-04-21|NS|EPI_ISL_18900594|NS
A/chicken/South_Korea/2230016/2022|2022-02-04|NS|EPI_ISL_19631541|NS
89
A/chicken/South_Korea/2130043/2021|2021-04-30|NS|EPI_ISL_18900591|NS
A/chicken/South_Korea/N23-054/2023|2023-05-15|NS|EPI_ISL_19631522|NS
A/chicken/South_Korea/N22-37/2022|2022-05-13|NS|EPI_ISL_19631513|NS
A/duck/South_Korea/N22-47/2022|2022-05-13|NS|EPI_ISL_19631528|NS
77
A/chicken/South_Korea/N22-43/2022|2022-05-13|NS|EPI_ISL_19631514|NS
A/chicken/South_Korea/N22-62/2022|2022-06-22|NS|EPI_ISL_19631516|NS
A/chicken/South_Korea/N22-63/2022|2022-06-22|NS|EPI_ISL_19631517|NS
A/chicken/South_Korea/N23-029/2023|2023-03-30|NS|EPI_ISL_19631521|NS
A/pigeon/South_Korea/N23-036/2023|2023-03-30|NS|EPI_ISL_19631559|NS
A/chicken/South_Korea/2330053/2023|2023-04-25|NS|EPI_ISL_18900595|NS
A/chicken/South_Korea/2330054/2023|2023-04-28|NS|EPI_ISL_18900596|NS
A/chicken/South_Korea/2330055/2023|2023-04-28|NS|EPI_ISL_18900597|NS
A/chicken/South_Korea/2330046/2023|2023-04-18|NS|EPI_ISL_18900593|NS
A/chicken/South_Korea/2330076/2023|2023-06-22|NS|EPI_ISL_18900599|NS
A/chicken/South_Korea/2330056/2023|2023-04-28|NS|EPI_ISL_18900598|NS
A/chicken/South_Korea/N22-142/2022|2022-09-01|NS|EPI_ISL_19631509|NS
A/duck/South_Korea/N22-122/2022|2022-09-01|NS|EPI_ISL_19631525|NS
A/duck/South_Korea/N22-121/2022|2022-09-01|NS|EPI_ISL_19631524|NS
A/chicken/South_Korea/N22-143/2022|2022-09-01|NS|EPI_ISL_19631510|NS
A/chicken/South_Korea/N23-058/2023|2023-05-15|NS|EPI_ISL_19631523|NS
87
A/chicken/South_Korea/N23-026/2023|2023-03-30|NS|EPI_ISL_19631520|NS
A/chicken/South_Korea/N23-010/2023|2023-01-13|NS|EPI_ISL_19631518|NS
A/chicken/South_Korea/N22-152/2022|2022-11-10|NS|EPI_ISL_19631511|NS
A/duck/South_Korea/N22-161/2022|2022-11-10|NS|EPI_ISL_19631526|NS
A/chicken/South_Korea/N23-015/2023|2023-01-13|NS|EPI_ISL_19631519|NS
